# Human Immunity and the Design of Multi-Component, Single Target Vaccines

**DOI:** 10.1371/journal.pone.0000850

**Published:** 2007-09-05

**Authors:** Allan Saul, Michael P. Fay

**Affiliations:** 1 Laboratory of Malaria and Vector Research, National Institute of Allergy and Infectious Diseases, National Institutes of Health, Rockville, Maryland, United States of America; 2 Biostatistics Research Branch, National Institute of Allergy and Infectious Diseases, National Institutes of Health, Rockville, Maryland, United States of America; AIDS Research Center, Chinese Academy of Medical Sciences and Peking Union Medical College, China

## Abstract

**Background:**

Inclusion of multiple immunogens to target a single organism is a strategy being pursued for many experimental vaccines, especially where it is difficult to generate a strongly protective response from a single immunogen. Although there are many human vaccines that contain multiple defined immunogens, in almost every case each component targets a different pathogen. As a consequence, there is little practical experience for deciding where the increased complexity of vaccines with multiple defined immunogens vaccines targeting single pathogens will be justifiable.

**Methodology/Principal Findings:**

A mathematical model, with immunogenicity parameters derived from a database of human responses to established vaccines, was used to predict the increase in the efficacy and the proportion of the population protected resulting from addition of further immunogens. The gains depended on the relative protection and the range of responses in the population to each immunogen and also to the correlation of the responses between immunogens. In most scenarios modeled, the gain in overall efficacy obtained by adding more immunogens was comparable to gains obtained from a single immunogen through the use of better formulations or adjuvants. Multi-component single target vaccines were more effective at decreasing the proportion of poor responders than increasing the overall efficacy of the vaccine in a population.

**Conclusions/Significance:**

Inclusion of limited number of antigens in a vaccine aimed at targeting a single organism will increase efficacy, but the gains are relatively modest and for a practical vaccine there are constraints that are likely to limit multi-component single target vaccines to a small number of key antigens. The model predicts that this type of vaccine will be most useful where the critical issue is the reduction in proportion of poor responders.

## Introduction

Chronic infectious diseases (e.g. malaria, tuberculosis, HIV) pose major challenges for new vaccines since these pathogens have evolved a variety of defense mechanisms that result in ineffective immunological responses to their constituent immunogens. Several strategies for trying to develop efficacious vaccines for these diseases include:

1. The use of genome and proteome projects to enable the systematic screening of all relevant pathogen proteins to identify the best immunogens, especially for bacterial [1] or parasitic diseases [2].
2. Improving the quantity or quality of the immune response by engineering the immunogen [3], [4] and through the use of more aggressive adjuvants. Although increasing understanding of the innate immune system [5] and the mechanisms of adjuvant action is likely to yield significant advances, the use of new adjuvants may be limited by safety or reactogenicity considerations [6].
3. The use of multiple immunogens. This is a strategy being pursued in a number of experimental vaccines. Although an obvious approach, there is surprisingly little experience with licensed human vaccines comprised of mixtures of defined immunogens that target a single pathogen. There are many human vaccines that contain mixtures of immunogens, but the different components usually target immunologically different pathogens, either different species or different serotypes of a single species (e.g. Pneumonococcal vaccines). Acellular pertussis vaccine is one multi-component, single target vaccine that contains two to five defined components [7] but even for this, there is debate as to whether the mixture is better than a vaccine based on pertussis toxin alone [8].

There are two distinct measures of the performance of a vaccine. 1) Vaccine efficacy is determined by recording the incidence of clinical episodes in a vaccinated group versus a control group over a defined time. This provides a measure of efficacy in the population based on the relative risk of contracting the disease. 2) The proportion of the vaccinated population that achieve a “protective” level of immunity e.g. for Hepatitis B, the proportion of the population that generate an anti-HbSAg antibody of 10 mIU per mL. For public health campaigns aimed at generating herd immunity to chronic infections, the distribution of individual risks is important, since poor responders may become the reservoirs of infection [9]. Although minimum protective levels are commonly assigned to vaccines, the basis for this assignment is often difficult [10]. Where immune responses correlate with subsequent protection, there is a continuous variation between immune response and the risk of disease, not an absolute cut off [9]–[11].

In practice, gains in efficacy by adding multiple immunogens will be balanced by the additional cost and complexity of manufacture, the need for a common adjuvant, the increased risk of adverse event as the immunogen load is increased, the possibility of chemical interactions between the components leading to inactivation of individual immunogens [12] and the problems of immunological interactions leading to poor immune response to one of more components. The vaccine complexity may be further exacerbated by the need in some existing and experimental vaccines to cover immunogenic diversity by inclusion of different serotypes of each immunogen. This cost of increasing the number of immunogens will depend on the delivery system employed: for protein based vaccines, fusion proteins have been used to deliver several immunogens as a single protein [13], [14]; DNA based vaccines as either naked DNA [15] or part of a viral deliver system [16] have been promoted, in part, on their ability to deliver complex mixes of immunogens.

To provide a basis for more rational design of multi-component vaccines, we have developed a mathematical model to investigate the potential gains in efficacy generated by combination vaccines. The model allows a comparison between the gains in efficacy from adding multiple immunogens with gains from more potent formulations or by choosing more efficacious immunogens and delineates the limited set of conditions where multi-component vaccines will be of practical importance.

## Methods

### Data Base

NBCI Pubmed (http://www.ncbi.nlm.nih.gov/entrez/query.fcgi?db = PubMed) was searched to find all references to “vaccine AND clinical Trial[publication type]” published from 1/1/1999 to 9/16/2006. The QUOSA Information Manager (http://www.quosa.com) [17] as used to download all PDF files accessible through the NIH electronic library. 1668 files were obtained.

These were searched within QUOSA to find the terms “reverse distribution” OR “reverse cumulative” anywhere within the text of these 1668 PDF files. This search found 66 references. These references containing reverse cumulative distributions [18] of immune response were selected since these have the potential to check summary information (geometric means and 95% confidence limit) against the actual distribution of antibody responses.

Antibody geometric means and 95% confidence limits were recorded for IgG levels, hemagglutination inhibition titers or neutralization titers for post primary vaccination series and for post boost antibody levels where the booster vaccine was not part of the initial vaccinations (e.g. for a 2, 4, 6 months infant immunization schedule followed by a 12 months boost, the geometric mean antibody level and confidence limits were recorded for months 7 and month 13 antibody levels). Antibody levels were not analyzed for pre-vaccination samples, samples collected part way through a vaccination series or for pre-boost samples. In all, 574 antibody distributions were recorded from 40 of the 66 papers. The most common reason for rejecting data from individual papers was the lack of quoted confidence intervals. Geometric mean antibody level and confidence intervals were recorded regardless of whether the paper described the corresponding reverse cumulative distribution. Generally the geometric mean antibody level and the upper and lower 95% limits were stated in the papers. In two cases, the quoted figures were internally inconsistent and these distributions were not included in the analyzed data set.

From the geometric mean antibody level, the 95% confidence interval of the means and the sample size, the standard deviations of the log transformed data were calculated, the 95% limits on distribution of the log transformed immune responses and the ratio of the upper to lower 95% limits of the non-transformed population distribution calculated (the fold range) and tabulated (Supplementary files Database S1 and Text S1). In principle, to calculate the standard deviation from the published confidence interval, it is necessary to know if the original authors used a t distribution or a normal distribution to calculate confidence intervals and this is often not cited in the publication. In practice, since most of the data sets have large numbers of subjects, this made little difference. In calculating the fold range, the median values were 64 and 68 on the assumption that all authors used a t distribution or a normal distribution, respectively. Thus in choosing representative values (9, 65 and 5000) of the fold range to use for modeling, we have picked rounded values that lie between the estimates from the two methods for the lower 2.5% quantile, median and upper 2.5% quantile, respectively.

These papers contained 408 usable post vaccination reverse cumulative distributions. They were not examined further if the distribution did not include the complete antibody range of subjects, if the number of subjects in the distribution was not recorded or if there were other factors missing that made interpretation impossible (e.g. the antibody scale was missing for the distributions in one paper). For each immunogen that was present more than once in the data base, a distribution was chosen where the complete reverse cumulative distribution was available, where the quality of the published distributions allowed a high quality digitization and curves with ranges and geometric mean antibody levels close to the center of the range for that immunogen. The published distribution was digitized from the PDF file, individual data points extracted, the geometric mean antibody and 95% confidence limits of the geometric mean antibody calculated and compared to the published values to check on the accuracy of the digitization. The distribution of the log transformed antibody data were graphically compared with a normal distribution using a Q-Q plot and numerically through the use of a Shapiro-Wilk test using the W statistic as a measure of departure from normality [19].

### Model

The model assumes that there is a monotonically increasing relationship between immune response and probability of protection, that there will be a range of responses occurring in the vaccinated population and that the responses to different immunogens in a vaccine will vary from being negatively to tightly positively correlated. In the model presented here, we assume that the immunity induced by each component is “additive”. Formally, we assume that the additive response conforms to Bliss independence [20], [21]; the probability of infection (or disease, depending on the vaccine) in the presence of an immune response to a mixture is the product of the probabilities of the infection in the presence each separate immune response. Thus if each of two immunogens reduces the risk of disease to 1/2, the combination will reduce the risk to 1/4 (i.e. 1/2×1/2).

The relative risk of the *i*th subject attributed to the *X_ij_* immune response to the *j*th immunogen was calculated using the Hill function [22].
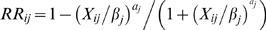
Where *β_j_* is the immune response required to give a *RR* of 0.5. In this formulation of the model, when *a_j_*>0, the risk decreases to zero as the immune response increases. The value *a_j_* = 1 was used for all simulations reported in this paper, but this fixing of *a_j_* = 1 does not hinder the generality of the model (see below).

The relative risk (RR) of the *i*th subject contracting disease following vaccination with a mixture of *n* immunogens is
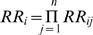
The efficacy of the vaccination in the population was calculated as 1-mean relative risk, where mean relative risk is calculated by taking the expectation over a population assuming the log transformed immune responses are normally distributed. The details of these calculations are given in the Supplementary file Text S1. The expectations were calculated for each of many different populations that each have different geometric means. For example, if *µ*_1_ is the mean of the log transformed and standardized immune response of the first component for a population (i.e., the mean of log_10_(*X_i_*_1_/*β*_1_)), then the geometric mean of that population is 10*^µ_*1*_^*. The standard deviation of the log transformed responses of a population is denoted *σ*_1_. In this paper for interpretability we use the “fold range”, which is a simple function of that standard deviation (specifically, 10^3.92σ_1_^), and the fold range is the ratio of the upper to lower quantiles of the middle 95% of each immune distribution (see Supplementary file Text S1 for derivation).

We established that the log transformed antibody level is approximately normal (see Results) and using the assumption that the distribution of antibody responses is a useful surrogate for the distribution of immune responses in general, the published 95% confidence intervals for the geometric mean immune response of the population were used to calculate and compare fold ranges in the 574 combinations of antigens and populations vaccinated in the assembled database. Since the antibody responses of trials are commonly quoted as geometric mean responses, we will use “geometric mean” in this paper but note that as the log antibody responses fit a normal distribution, the geometric mean of a population is also its median.

By properties of the normal distribution, we can show the generality of the model. Specifically, the mean efficacy from the model with *a_j_* = 1, mean = *µ_j_*, and standard deviation = *σ_j_* is equivalent to the mean efficacy from the model with *a_j_* = *a*, mean = *µ_j_/a* and standard deviation = *σ_i_/a*, for any *a>*0

The increased immunogenicity required for a single component vaccine to match the protection afforded by a mixture was calculated by comparison of the efficacy or % protected values for the mixture with the mean efficacy vs. immune response or % protection vs. immune response relationships for a single component vaccine.

For modeling mixtures of immunogens that individually generate different levels of protection, the most active immunogen as judged by the average level of efficacy in the population, was used as the comparator immunogen and the ratio its geometric mean to those of the 2^nd^ or 3^rd^ immunogens was kept constant as the geometric mean immune response to the first antigen was varied. For example, if the second component has 10% of the geometric mean of the first component, the geometric mean antibody to the second antibody will be 0.1 unit when the geometric mean antibody to the first immunogen is 1.

An R package (Supplementary file Software S1. See Supplementary file Text S1 for instructions on how to download a copy of the package and on its use) was developed to calculate the efficacy of mixtures, the proportion protected and the increase in immune levels required for a single component vaccine to match a mixture. The R package is the definitive version of the model. A simplified version of the model is also available as a Microsoft Excel spreadsheet (Supplementary file Spreadsheet S1.) This needs to be downloaded and decompressed prior to use. Instructions for use are contained within the spreadsheet.

## Results

### Distribution of human immune responses to conventional vaccines

A database of published human immune responses to conventional vaccines was used for assigning parameters to the model. Thirty of the published reverse cumulative distributions [18] of the vaccine responses to individual immunogens were used to determine the distribution of the antibody responses. In all cases, as judged by the shape of the q-q plot, the distribution of the log transformed data were close to normal (mean Shapiro-Wilk [19] W = 0.96). 13/30 had a significant departure (p<0.05 without correcting for multiple comparisons) from normality. However, even for these, the Shapiro-Wilk coefficient was > = 0.88 suggesting that the deviation from normal was small. For the vaccine trials that gave the greatest departures from normality (a hepatitis A vaccine [23] and a *S. pneumoniae* type 19F vaccine [24] trial), the distribution of the log transformed data from other 3 other trials examined with each of these vaccines were not significantly different to a normal distribution (data not shown).

The observed fold range depends on the immunogen. Over the whole set of data analyzed, the median fold range was approximately 65 (95% of the observed fold ranges were approximately between 9 to 5,000 fold depending on the assumptions used to analyze individual data–see “Materials and Methods” for details).

**Figure 1:**
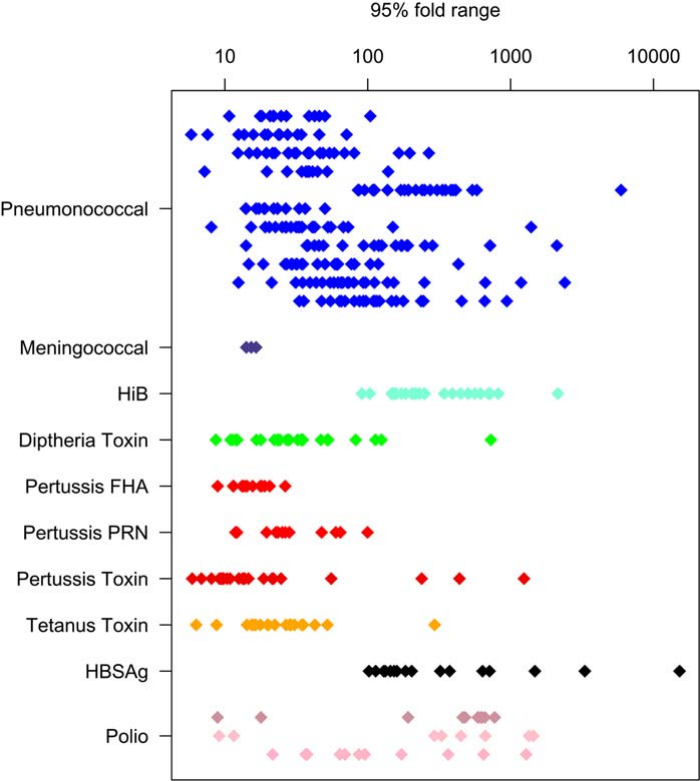
The fold range of the immune response reported for 396 immunogens in vaccine trials in infants. Each point is the ratio of the estimated 97.5^th^ percentile antibody response to the 2.5^th^ percentile response (i.e. the 95% range) in the post vaccination sera. Most vaccine trials contained several immunogens. Eleven serospecificites are reported for pneumonococcal vaccines. The order from top to bottom is serotypes 1, 3, 4, 5, 6B, 7F, 9V, 14, 18C, 19F and 23 F. The four meningococcal vaccine results are from the only infant trial in this data base and are a single determination of each of the A, C, W173 and Y specificities. Polio values are ranges for neutralizing activity for Polio 1, 2 and 3, respectively, from left to right. References to the individual trials are contained in supplementary file Database S1.

Protein toxoid responses (diphtheria, tetanus and pertussis) tended to have smaller fold ranges that other vaccines, but this was not specific to protein immunogen since the fold range of responses to the Hepatitis B was the largest observed. Even for conjugate vaccines that shared the same carriers, the fold range of responses varied significantly. For example, in Fig. 1, there are 23 determinations of the fold range of response to *S. pneumoniae* serotype 6B and 9V in paired trials where the same carrier is used in each pair. In these studies, serotype 6B has a significantly higher fold range than 9B (P<0.0001, Z = 4.18, Wilcoxon signed rank test).

For a single vaccine, there is considerable variation in the measurement of that fold range in different trials. For 5 of 18 immunogens tested in infants, there was a significant inverse correlation between the geometric mean immune response and fold range (Table 1), but even where significant, this only accounts for part of the variation. The database predominantly reports vaccine trials in infants. Where the same vaccine was used in older age groups the fold range usually increased with age, although whether this is due to prior exposure or a direct effect of aging could not be assessed. In one case, a wider fold range was associated a measurement of the response prior to the peak response[25].

**Table 1:** Spearman rank correlation between geometric mean antibody responses and fold range of antibody responses in vaccine trials in infants.

Vaccine	Immunogen	No. of trials	Spearman rank correlation
Pneumonococcal	1	13	−0.02
	3	13	−0.35
	4	23	0.10
	5	13	0.08
	14	23	−0.42*
	18C	23	−0.30
	19F	23	0.20
	23F	23	0.04
	6B	23	0.21
	7F	13	0.24
	9V	23	−0.37*
HiB	PRP	28	−0.37
Diphtheria	DT	30	−0.11
Pertussis	FHA	13	−0.19
	Pertactin	12	−0.62*
	PT	22	−0.45*
Hepatitis B	HbS	13	−0.44*
Tetanus	TT	20	−0.04

* P 0.017 to 0.045

On the basis of these results, we chose the underlying distribution for modeling the efficacy of combination vaccines as a normal distribution on the log immune response such that the fold range is 65 on the untransformed responses.

### Mixtures of independent immunogens with equal individual efficacy

**Figure 2:**
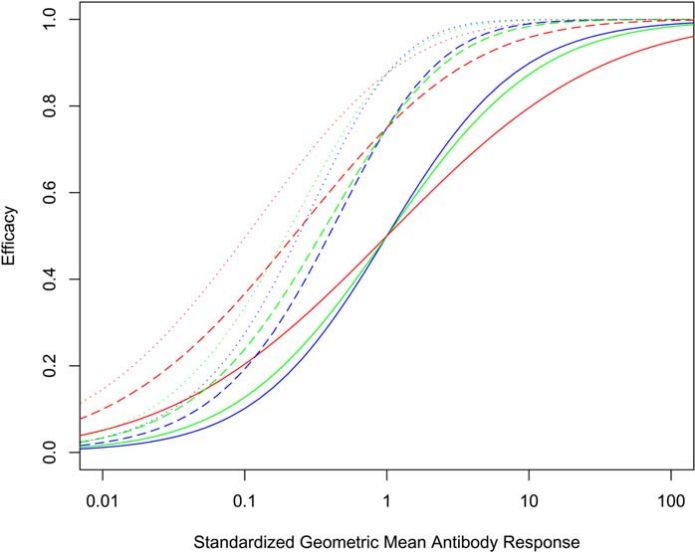
Model prediction for the efficacy of single component vaccines (solid line), two component vaccines (dashed lines) and three component vaccines (dotted lines) as a function of the mean immune response elicited in the population to each component. This data set assumes that each component contributes equally to the efficacy, the immune responses to the individual immunogens are not correlated and that the log transformed distribution of the immune response is normal with a fold range of 9 (blue), 65 (green) and 5,000 (red).

Fig. 2 illustrates the predicted efficacy for vaccines with one, two or three immunogens as a function of the geometric mean immune response in the population and for immunogens that give a narrow, median or wide fold range of immune responses. In all models, the immune responses for each component are scaled so a person with an immune response of 1 unit would have a 50% decrease in risk. For this model, we assume that for vaccines with two or three immunogens, each immunogen induces the same geometric mean response in the population; has the same fold range of immune responses, and has no correlation between the immune responses to the component immunogens. For vaccines with a single component, even though the risk of infection to an individual is determined by the individual immune response, the efficacy in the population depends on both the geometric mean and the fold range of immune responses in the population. Measures that increase the average immune response (e.g. more active adjuvants or altered vaccination regimens) have a bigger impact on efficacy for vaccines that have a narrow fold range of responses, than vaccines that have a broad fold range.

As expected, addition of a second or third vaccine component increases the efficacy of the vaccine. There are two effects: an increase in efficacy for any given immune response; a steeper antibody: efficacy response for the mixtures.

**Figure 3:**
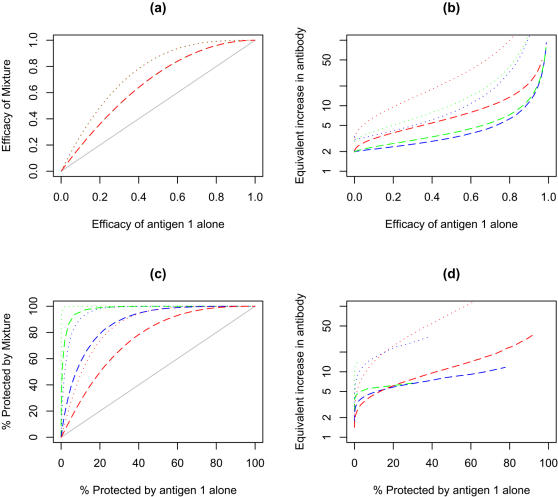
Effect of the diversity of immune response on efficacy and percentage of the population protected by two component (dashed lines) and three component vaccines (dotted lines) for vaccines using the same model parameters as Fig. 2 where each component has the same average immunogenicity. (a) The efficacy of a two or three component (grey solid line) as a function of the average efficacy of single component vaccines. Diversity of the immune response has no impact on the efficacy of the mixed vaccine so the curves for the three component vaccines (dotted upper lines) coincide as do the curves for the two component vaccines (dashed middle line). (b) Relative immunogenicity that would be needed with a single component vaccine to match the efficacy of a two or three component vaccine as a function of the average efficacy of single component vaccines. The relative immunogenicity is the ratio of the immune response required in a single component vaccine to the immune response of the best immunogen in a multi-component vaccine with similar efficacy (c) Comparison of the percentage of the population protected (relative risk of 0.1 or less) of a two or three component vaccine to the percentage protected with a single component vaccine (grey solid line) as a function of the percentage protected with single component vaccines of varying efficacy. (d) Relative immunogenicity that would be required with a single component vaccine to match the percentage protected with a multi-component vaccine as a function of the percentage protected with single component vaccines of varying efficacy. The relative immunogenicity can only be determined over the part of the range where <100% of the population given a mixed vaccine is protected.

For developing vaccine design strategies, it would be useful to predict the relative impact of different vaccine strategies. Using a single immunogen vaccine as the standard, the efficacy of a mixture as a function of the efficacy of a single immunogen vaccine has been plotted (Fig. 3a) or the increase required in immune response from a single immunogen vaccine to match the increased efficacy of a mixture (“relative immunogenicity”, Fig. 3b). In both cases, the outcomes have been plotted over a range of efficacies for the single immunogen comparator vaccine.

The efficacy of the mixture does not depend on the fold range of responses for the individual immunogens (Fig. 3a). However, the relative immunogenicity depends on both the geometric mean and fold range of the immune responses of the individual components.

The gains by mixing two or three immunogen are relatively modest: For example for a single immunogen vaccine that gives an average efficacy in the population of 50% and has a fold range of 65 fold, adding a second similar immunogen would increase the overall efficacy to 75%. The model predicts that this gain is equivalent to a four fold increase in immunogenicity for a single component with larger gains for vaccines that give a greater fold range in responses.

By contrast, mixtures of immunogens are predicted to have large impacts on the proportion of poor responders in the population and this impact is highly dependent on the fold range induced by the vaccine. In Fig. 3c the proportion protected with a mixture is plotted as a function of the proportion protected by a single immunogen. The data for this figure assume that the “protected” individuals have >90% decreased probability of disease. Qualitatively similar results are obtained with other “protection” thresholds (data not shown). A large increase in immunogenicity is required for a single vaccine to match the percent protection induced by a mixture and is largely independent of the fold range (Fig. 3d).

### Mixtures of independent immunogens with unequal individual efficacy

**Figure 4:**
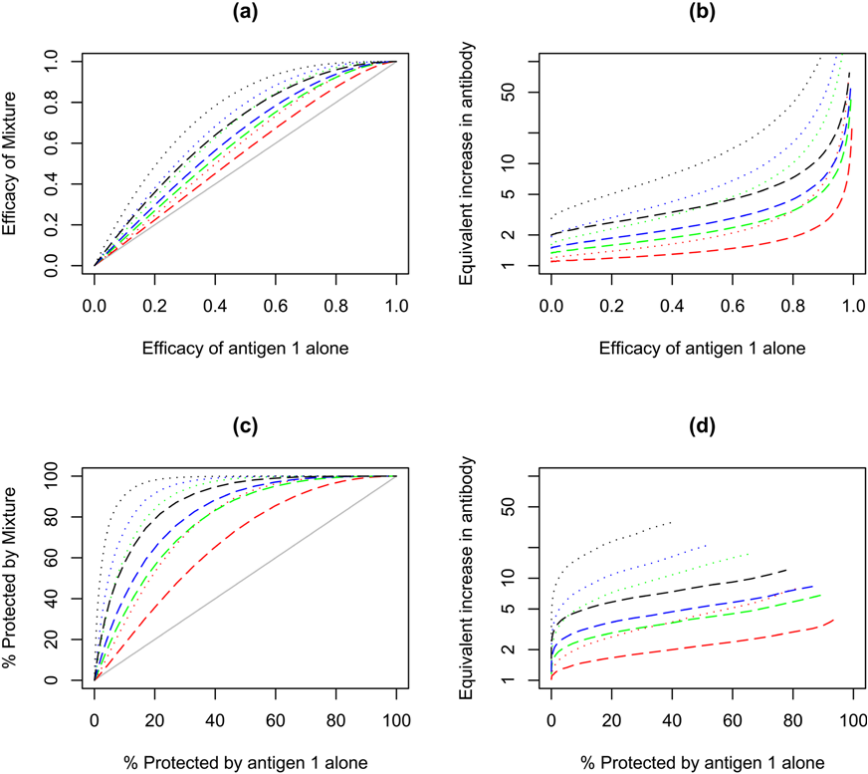
Effect using immunogens that elicit different levels of immunity in a two component (dashed lines) or three component (dotted lines) vaccines on efficacy and the percent population protected. Model assumes that the diversity of the immune response generated by each component is similar (fold range is 65 fold) and that the immune response to the individual components is not correlated. Vaccines contain a second or third immunogen that generate 1/10 (red), 1/3 (green), 1/2 (blue) or equal geometric mean immune responses (black) to the geometric mean of the first component. Other details are described in the Figure 3 legend.

The efficacy of mixtures of immunogens that when used individually give geometric mean responses that are 10%, 33.3% and 50% of the geometric mean response of the most efficacious component, are plotted in Fig. 4a as a function of the efficacy of the best component acting alone and their relative immunogenicity in Fig. 4b. As the efficacy of the second or third components drops compared to the efficacy of the most active component, the gains obtained by mixing on overall efficacy (Fig. 4a) or the proportion protected (Fig 4c) decrease substantially as does the relative immunogenicity of a single component vaccine that would have been required to match the efficacy of a mixture (Fig. 4b) or the proportion protected (Fig. 4d).

### Mixtures of immunogens with correlated immune response and with equal individual efficacy

**Figure 5:**
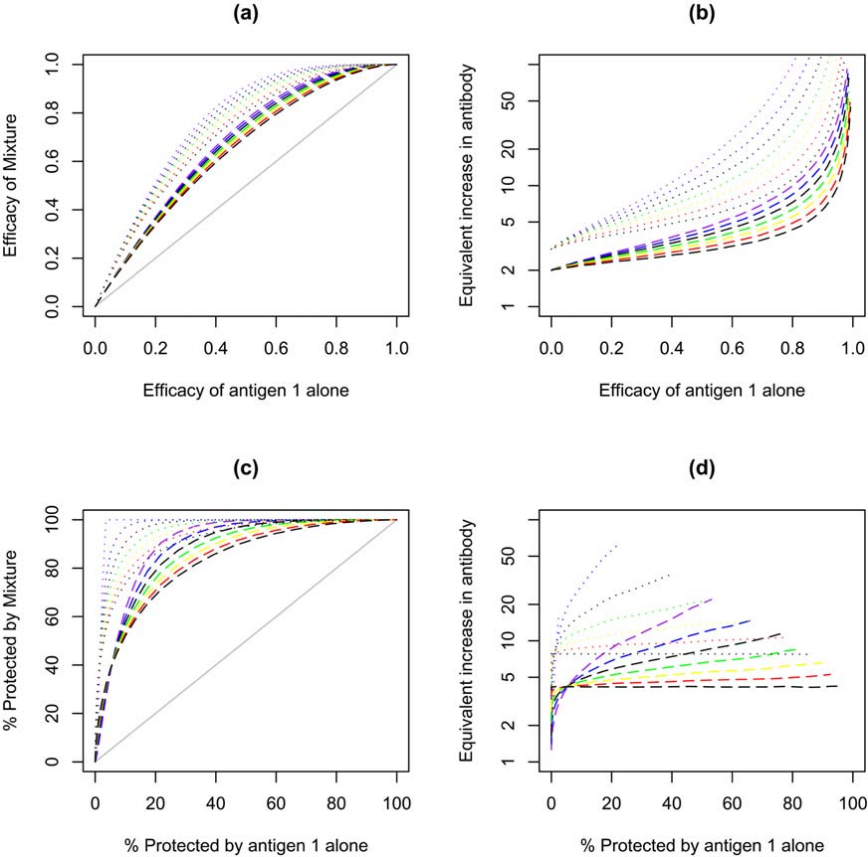
Effect of the correlation of immune responses to the immunogens in a two component (dashed lines) or three component (dotted lines) vaccine on efficacy and the percent population protected. Model assumes that the diversity of the immune response generated by each component is similar (fold range is 65 fold) and that the level of the immune response to the individual components is similar. Immune responses have a correlation coefficient (r) of 1 (lower black line), 0.75 (red), 0.5 (yellow), 0.25 (green), 0 (upper black), −0.25 (blue) and −0.5 (purple). Other details are described in the Figure 3 legend.

Correlation of the immune response of the components in a mixture is predicted to have an impact on both the overall efficacy of the mixture and on the proportion of people protected by the mixture (Fig. 5a to 5d). Immunogens that induce independent immune responses giving greater increases in efficacy in the mixture compared to immunogen that give highly positively correlated immune responses. Theoretically, immunogens may give negatively correlated responses. A negative correlation enhances the efficacy and proportion protected compared to independent responses.

## Discussion

This study predicts that under optimum conditions, useful gains in efficacy and in the proportion of responders in a community can be obtained through the use of vaccines containing mixtures of immunogens targeting a single pathogen. This analysis suggests that the key factor for multi-component vaccines is the quality of the immunogens and the potency of the formulation. Especially for strategies aimed at improving the average efficacy of a vaccine, the gains from mixing several immunogens are relatively small and are similar to the gains obtained by increasing immunogenicity by approximately 3 to 5 fold.

In the context of developing vaccines, a 3 to 5 fold difference in immunogenicity is seen in different versions of some current vaccines. For example, in a large trial of infants vaccinated with three different acellular pertussis vaccines there was a 3 fold difference between the geometric mean anti-pertussis toxin level and a 5 fold difference between the anti-filamentous hemagglutin levels between two of the vaccines [7]. Thus, creation of multi-component vaccines is unlikely to be justifiable until it is known that careful optimization of formulation results in a vaccine that just fails to achieve the necessary efficacy or the proportion protected.

Depending on the immunogen, addition of adjuvants can make a large difference. An extreme example is the 10,000 fold difference recorded between the antibody response to a non-adjuvanted hepatitis B vaccine and to the same immunogen after the addition of the AS02 adjuvant [26]. Comparison of the currently used alum adjuvanted hepatitis B vaccine with the same vaccine after the addition of the immunostimlatory oligonucleotide CPG 7909 gives a 10 fold increase in antibody after two vaccinations [27]. A 10 fold increase in antibody response would give a comparable or greater increase in efficacy than the increase in efficacy predicted by this model for adding a second or third immunogen.

Two other immunogen related factors impact the predicted gains. The gain in efficacy from generating a mixture does not depend on the diversity of the immune response. However, a larger increase in antibody is required to give a similar increase in efficacy in a vaccine that has a highly diverse immune response compared to a more homogeneous response. When compared to other methods of improving efficacy, mixtures may be more attractive for immunogens that generate a diverse response. On the other hand, with immunogens that give a smaller variation in antibody responses, larger gains in the proportion of responders is possible with mixtures. The correlation of the immune responses between different immunogens also influences the gains: for the additive model considered here, mixtures of immunogens that give a highly correlated response give smaller gains in both efficacy and in the proportion of the community protected than immunogens with independent responses.

The model predicts that use of mixtures may be more useful for decreasing the proportion of poor responders in a community than in increasing the overall efficacy. If the increase in efficacy or the proportion protected is expressed in equivalent immunogenicity, the gain in the proportion protected is approximately twice the gain in efficacy. For example, the gain in efficacy by mixing two immunogens each of which give a mean 50% efficacy and for which the responses are independent, is equivalent to improving the immunogenicity of a single component by 3.8 fold (Fig. 3b, binary mix, fold range 65) with a corresponding increase in efficacy from 50 to 75%. By contrast, the gain in proportion protected with the same combination that gave 50% protected, is equivalent to increasing the immunogenicity of a single component by 8.25 fold with a corresponding increase in proportion protected from 50 to 97.6%.

The gains in the proportion protected, are highly dependent on the correlation between the immune response (Fig. 5d). If there is a low correlation between the immune responses, then it is likely that a person who is a poor responder to one immunogen, will be at least an average responder to another immunogen, and therefore will be protected. If the responses are highly correlated, then in this model a person who is a poor responder to a single immunogen will still receive some gain from a mixture, (overall response at least as good as doubling the antibody level), but will still be a relatively poor responder overall.

Using the assumption of an additive response, for using multi-component to either boost overall efficacy or the proportion of people protected, a major limitation is the need to have immunogens that individually give similar efficacies. It seems unlikely that once the best immunogen has been found for a particular pathogen, and the immunogenicity of the vaccine formulation using that immunogen optimized, that there would be multiple other immunogens that would give similar protection. Therefore, most practical vaccines are unlikely to contain more than two or three immunogens targeting a single pathogen, especially as many vaccines being developed may also require multiple variants of each immunogen to overcome antigenic polymorphisms in the targets. Furthermore, since gains in efficacy or percent protected from making a binary or even tertiary mixture are relatively modest, it would also make it unlikely that mixtures of components that are poor immunogens, e.g. fail to generate a significant biological response in a Phase 2 trial, would form a useful vaccine unless there was some major synergistic response that was undetectable in single antigen trials.

The development of this model highlighted several areas where there was little available information on human responses to vaccines in general or to mixed immunogens in particular. There are few reports on the details of the relationship between the strength of a vaccine induced response and the subsequent relative risk of disease. This is a difficult relationship to measure since it requires a high disease incidence or large group sizes and may contain substantial error. Although different relationships have been proposed in individual studies, all are broadly compatible with the simplest relationship, the Hill function, chosen for this model. The use of the Hill function chosen for this study potentially allows a wide variety of response/protection relationships to be modeled and this may be important in modeling individual vaccines where the relationship between immune response and protection is known or measured.

Given the large number of trials published with multi-component vaccines in which individual immune responses to the component immunogens have been measures (e.g. most of the trials referenced in the data base used for this study, supplementary file Database S1), there is surprisingly little published data on the correlation between the human immune response to different antigens in a multi-immunogen vaccine. As a result, we have chosen to investigate the range of possible correlations rather than chose literature values.

Immunological interference is a concern in multi-component multi-target vaccines where the immune response to one component decreases the response and thus the protection to a different component. Reflecting this concern, the FDA in its guidance for industry for the evaluation of combination vaccines emphasizes the importance of non-inferiority tests to show that the immune response to each component of a vaccine is not less than the response to the same component in a single component vaccine[28], [29]. However this guidance is specifically for multi-target vaccines (either multiple diseases or multiple serotypes of a single pathogen species) and not for the multi-component, single target vaccines considered in this study.

Immune interference is less likely to be a problem for multi-component vaccines targeting a single organism. In multi-target vaccines, a strong response to one immunogen could decrease an otherwise marginal response to a second immunogen and render the subject vulnerable to infection with the pathogen corresponding to the second immunogen. In single target vaccines, the major problem with antigenic interference would only occur in the unlikely case of a weak response to one immunogen substantially decreasing the strong response to a second immunogen. In any case, for a multi-component, single target vaccine, the critical test is if the combination gives a better efficacy than the individual immunogens, and this could occur even if the immune response to the individual components was weaker in the combination than individually. This is not covered by the existing FDA guidance. Despite the abundance of data on human trials for multi-component, multi-target vaccines, the development and regulatory considerations for multi-component, single target vaccines remains to be fully developed.

This model assumes that protection from the immune response to each component is additive as this is the simplest assumption for how the individual responses will combine to determine the overall efficacy of the vaccine in an individual. Theoretically, either a synergistic effect, where the overall efficacy was greater than predicted, or a less than additive response could apply to specific combination vaccines. One extreme case of the latter situation was considered in developing this model: where the efficacy of the mixture was equal to the efficacy of the best component (i.e. no additive response at all). Under these conditions, the combination could give substantial improvement compared to the individual vaccines, but this increase was critically dependent on the correlation between the responses to the individual components: the increased efficacy required a low correlation between individual responses (results not shown).

For all examples analyzed in this study, antibody level is used as a measure of immunity. However, the model is general and other measures, e.g. levels of cytotoxic T-lymphocyte (CTL) responses could be substituted with some provisos, including a consideration of how protective responses to different antigens are correlated in the population and whether the additive model applies. The correlation of protective responses may be particularly important for peptide based CTL vaccines containing a restricted number of epitopes that are in turn HLA restricted. However, we would expect the same general results: the gains in adding multiple antigens will be relatively modest and only achieved where each individual antigens elicits a broadly similar protective effect and that the complexity of a vaccine may be further limited by the need to include multiple variants of each antigen to protect against antigenic polymorphisms.

In conclusion, after optimizing immunogen choice and formulation, significant gains in efficacy and the proportion of the population protected by a vaccine may be obtained by addition of further antigens targeting the same pathogen. While the gains may be significant on a public health level, they are likely to be relatively small. In one sense this makes decisions to proceed with a multi-component vaccine simpler: unless each of the component antigens have demonstrable activity, it is unlikely that the mixture will be useful. On the other hand, demonstrating the benefit of a mixture over a single component vaccine may be difficult and require careful trial design. In some cases, justification for the mixture may depend only on a theoretical consideration of the efficacy of the individual components and rely on procedures such as the model presented in this paper.

## Supporting Information

Text S1 Multi-component, single target vaccine R program derivation and instructions. This file contains further details of the derivation of the model, instructions for downloading and installing the definitive version of the model written in the R statistical programming language, instructions for using the model, and examples of input and output from the model.
(0.45 MB PDF)

Database S1 Database of antibody responses from published trials. This file contains the dataset of the magnitude and range of responses.
(0.16 MB XLS)

Spreadsheet S1 Multi-component, single target vaccine Excel spreadsheet. This is a version of the model presented as an Excel spreadsheet and designed for a simpler user interface than the R program version. It generates a more restricted range of output compared to the R version. This file must be downloaded and decompressed before use. Instructions for use are included in the spreadsheet.
(4.23 MB ZIP)

Software S1 Multi-component, single target vaccine R program software package. The R package containing the model. Instructions for unzipping and installing this program are contained in the supplementary file Hbimdetails.pdf
(0.60 MB ZIP)
