# Supplementary material for: Human Immunity and the Design of Multi-Component, Single Target Vaccines
Source: PLoS One. 2007 Sep 5;2(9):e850. doi: 10.1371/journal.pone.0000850 (PMC1952173; doi:10.1371/journal.pone.0000850)
Supplement: Text S1 — Multi-component, single target vaccine R program derivation and instructions. This file contains further details of the derivation of the model, instructions for downloading and installing the definitive version of the model written in the R statistical programming language, instructions for using the model, and examples of input and output from the model. (0.45 MB PDF) [file pone.0000850.s001.pdf]

Mathematical and Computational Supplement to "Multicomponent,single target vaccines: benefits and limitations for practical vaccine design derived from mathematical models"

by Allan Saul and Michael P. Fay

June 22, 2007

Summary

This supplement gives the details of the calculations that produce the figures in the paper. This document is part of an R package called `hbim` which contains all the data and software needed to reproduce the calculations for the figures of the paper.

1 Introduction

The document is part of an R package that allows reproducible research as discussed in Gentleman (2005) and Gentleman and Temple Lang (2007). With this package, called `hbim`, a researcher can redo all the calculations done to produce the figures in the main paper. Further one can extract the R computer code used to create those Figures and modify it as desired.

Here is an outline of this document. The main part of the document gives the overall details of the calculations in mathematical notation, while Appendix A gives the details of the computer programs used in R package. In Section 2 we define the 95% range and 95% fold-range. This is a simple function of the standard deviation of the log of the transformed antibody responses, denoted σ. In Section 3 we give the algorithm for estimating σ (the standard deviation of the log transformed responses) from the 95% confidence interval of the mean. In Section 4 we show how we calculate the relative risk and the expected relative risk. In Section 5 we show how we calculate the percent protected from this model.

Appendix A gives details of how the calculations were done, and information on how to redo the calculations in the R programming language using the `hbim` package. R is an open source freeware statstical

programming language (R Development Core Team, 2007). Within Appendix A we reproduce many of the figures of the main paper. Appendix B gives some basic properties of the lognormal distribution.

2 Definition of 95% Range

Let $y_i = \log_{10}(x_i)$, where x_i is the antibody level in the ith subject. Let Y_i be the random variable associated with y_i. We assume that the Y_i are independently normally distributed with mean θ and variance σ^2. Thus, $\frac{Y_i-\theta}{\sigma}$ is distributed standard normal. Let $\Phi^{-1}(q)$ be the qth quantile of the standard normal distribution. Thus,$Pr[\Phi^{-1}(.025) \leq \frac{Y_i-\theta}{\sigma} \leq \Phi^{-1}(.975)] = .95$, and under this model, we are 95% sure that a random log transformed antibody level is between $\sigma\Phi^{-1}(.025)+\theta$ and $\sigma\Phi^{-1}(.975)+\theta$. The 95% range on the log transformed scale is then

$$95\% \text{ Range} = \sigma\left\{\Phi^{-1}(.975) - \Phi^{-1}(.025)\right\} = 3.92\sigma.$$

On the untransformed scale the 95% range is the ratio of the upper to lower 95% confidence limits, (also known as the *95% fold-range*)

$$\frac{10^{\sigma\Phi^{-1}(.975)+\theta}}{10^{\sigma\Phi^{-1}(.025)+\theta}} = 10^{\sigma\left\{\Phi^{-1}(.975)-\Phi^{-1}(.025)\right\}} = 10^{3.92\sigma}.$$

3 Estimating the Standard Deviation of the Log Transformed Responses

As before let $y_i = \log_{10}(x_i)$, where x_i is the antibody level in the ith subject. Suppose there are n subjects measured. The usual unbiased estimator of the variance (i.e., standard deviation squared) is

$$s^2 = \frac{1}{n-1}\sum_{i=1}^{n}(y_i - \bar{y})^2.$$

where $\bar{y} = \frac{1}{n}\sum_{i=1}^{n} y_i$ is the mean of the y_i values. In the literature often this unbiased estimate is not given, but the 95% confidence interval is given. The usual 95% confidence interval based on the log scale calculated by the standard formula is based on either the t-distribution or the normal distribution, i.e., is either,

$$\bar{y} \pm \frac{s}{\sqrt{n}} t_{n-1}^{-1}(.975)$$

where $t_{n-1}^{-1}(q)$ is the qth quantile of the t distribution with n-1 degrees of freedom, or is

$$\bar{y} \pm \frac{s}{\sqrt{n}} \Phi^{-1}(.975)$$

where $\Phi^{-1}(q)$ is the qth quantile of the standard normal distribution (see e.g., Casella and Berger, 2002, p. 429). Note that $\lim_{n\rightarrow\infty} t_{n-1}^{-1}(q) = \Phi^{-1}(q)$ for all q. Thus, in practice it may not matter too much which is used. Here are some values for $t_{n-1}^{-1}(.975)$ for different values of n:

	$n=10$	$n=20$	$n=50$	$n=100$	$n=200$	$\lim_{n\rightarrow\infty}$
$t_{n-1}^{-1}(.975)$	2.228	2.086	2.009	1.984	1.972	1.960

Given the 95% confidence interval (L,U) we can solve for s. When the t-distribution was used for the confidence interval can find s by:

$$s = \frac{\sqrt{n}\,(U-L)}{2t_{n-1}^{-1}(.975)} \quad (1)$$

Similarly when the normal distribution was used,

$$s = \frac{\sqrt{n}\,(U-L)}{2\Phi^{-1}(.975)} = \frac{\sqrt{n}\,(U-L)}{3.92} \quad (2)$$

Since the confidence interval by the t-distribution is more common, we used that for all the calculations in this document.

4 Calculating Mean Relative Risk

We use a Hill function for relative risk for a single antibody concentration. Let the antibody level for the ith subject at the jth immunogen be x_{ij}, then the associated relative risk is

$$RR_{ij} = \frac{1}{1+\left(\frac{x_{ij}}{\beta_j}\right)^{a_j}}$$

where β_j is the antibody required to give a relative risk of 0.5, and a_j is a slope parameter. Let $d_{ij} = x_{ij}/\beta_j$ denote the antibody level standardized by β_j, so that $d_{ij} = 1$ is the antibody required to give a RR of .5, $d_{ij} = .5$ is half that amount, etc. Let $\beta_j^* = \log_{10}(\beta_j)$ and $d_{ij}^* = \log_{10}(d_{ij})$. Further, as above, let $y_{ij} = \log_{10}(x_{ij})$. Then we can write RR_{ij} as

$$RR_{ij} = \frac{1}{1+10^{a_j\left(y_{ij}-\beta_j^*\right)}}$$

If we assume that $Y_{ij} \sim N(\theta_j, \sigma_j^2)$ then $D^*_{ij} = \log_{10}(X_{ij}/\beta_j) = Y_{ij} - \beta^*_j \sim N(\theta_j - \beta^*_j, \sigma_j^2)$, and we can use the same σ_j^2 for the distribution of D^*_{ij} as was used for the distribution of Y_{ij}. So the mean of D^*_{ij} is $\theta_j - \beta^*_j$ and for a sample of D_{ij} values, the expected geometric mean is $10^{\theta_j - \beta^*_j} = 10^{\theta_j}/\beta_j$. For example, Figure 2 of the paper plots $10^{\theta_j - \beta^*_j}$ on the horizontal axis. Note that $10^{\theta_j - \beta^*_j}$ is not the expected value of D_{ij} (see Appendix B).

Based on the Bliss independence model (Greco, et al, 1995), The RR for the ith subject with J antigen components is

$$RR_i = \prod_{i=1}^{J} RR_{ij}. \tag{3}$$

In order to account for possible correlation between antibody responses with different antigens we model the D^*_{ij} as multivariate normal. Let $\mu_j = \theta_j - \beta^*_j$ so that

$$\begin{bmatrix} D^*_{i1} \\ \vdots \\ D^*_{iJ} \end{bmatrix} \sim N(\mu, \mathbf{V}) = N\left(\begin{bmatrix} \mu_1 \\ \vdots \\ \mu_J \end{bmatrix}, \begin{bmatrix} \sigma_1^2 & \rho\sigma_1\sigma_2 & \cdots & \rho\sigma_1\sigma_J \\ \rho\sigma_1\sigma_2 & \sigma_2^2 & \cdots & \rho\sigma_2\sigma_J \\ \vdots & & & \\ \rho\sigma_1\sigma_J & \rho\sigma_2\sigma_J & \cdots & \sigma_J^2 \end{bmatrix} \right)$$

where ρ is the correlation between any two antigen responses on the same subject, and μ is the mean vector, and $\mathbf{V}$ is the covariance matrix. Let the probability density function of the multivariate normal distribution with mean μ and covariance $\mathbf{V}$ evaluated at the J-dimensional point $\mathbf{t}$ be $\phi(\mathbf{t}, \mu, \mathbf{V})$. Then the expected RR_i over the whole population can be calculated by the J dimensional integral,

$$E(RR_i) = \int_{-\infty}^{\infty} \cdots \int_{-\infty}^{\infty} \left(\prod_{j=1}^{J} \frac{1}{1 + 10^{a_j t_j}} \right) \phi([t_1, \ldots, t_J], \mu, \mathbf{V})\, dt_1\, dt_2\, \cdots dt_J \tag{4}$$

We can use either numberic integration or integration by simulation (see Appendix A for details of which method is used for which integral). For a discussion on the accuracy of the integration by simulation, see the end of Section 5.

5 Calculating the Proportion Protected

Assume that RR_i is given by equation 3. Suppose we consider a subject protected if $RR_i \leq r_p$. In our paper we use $r_p = 0.10$.

If there is only one antigen then a subject with antibody level t_1 is protected whenever,

$$\frac{1}{1+10^{a_1 t_1}} \quad \leq \quad r_p$$

which implies protection when $t_1 \geq \tau$, where

$$\tau = \frac{1}{a_1} \log_{10}\left(\frac{1-r_p}{r_p}\right).$$

So the proportion protected in the population will be

$$PP(r_p) = \int_{\tau}^{\infty} \phi(t_1, \mu, \mathbf{V})\, dt_1 = 1 - \Phi(\tau, \mu, \mathbf{V}) \tag{5}$$

where $\Phi(\mathbf{t}, \mu, \mathbf{V})$ is the cumulative normal distribution with mean μ and variance $\mathbf{V}$.

If there are two antigens then if the ith subject has antibody levels t_1 and t_2, that subject is protected whenever $PP_i = 1$, where

$$PP_i \quad = \quad I\left\{\left(\frac{1}{1+10^{a_1 t_1}}\right)\left(\frac{1}{1+10^{a_2 t_2}}\right) \leq r_p\right\}$$

where $I(A) = 1$ when A is true and 0 otherwise. For J antigens, the expected proportion protected over the whole population can be calculated by the J dimensional integral,

$$E(PP_i) = \int_{-\infty}^{\infty} \cdots \int_{-\infty}^{\infty} I\left\{\prod_{j=1}^{J} \frac{1}{1+10^{a_j t_j}} \leq r_p\right\} \phi([t_1, \ldots, t_J], \mu, \mathbf{V})\, dt_1\, dt_2 \cdots dt_J \tag{6}$$

Since this integral is not smoothly changing, the numeric integration is not straightforward. An alternative is to integrate by simulation. In this case, since PP_i is a binary random variable, we can use the properties of binomial random variable to get a bound on the integration. Here and in the main paper we use 5e+05 simulations to estimate these integrals. If we do 5e+05 simulations, then we are 99.9% sure that the simulated mean will be within ± 0.001163 of the true value. This comes from the usual normal theory confidence interal, which is $mean \pm \frac{\Phi^{-1}(.9995)P(1-P)}{\sqrt{5e+05}}$, except we set P, the unknown true value of $E(PP_i)$ equal to $P = .5$ which maximizes the width of the confidence interval. If the true value of P is close to 0 or 1 then the confidence interval will be tighter; for example, if our estimate of P was .01 then we would be 99.9% sure that the mean would be within $\pm 4.6e-05$.

Note that similar statements about the accuracy of the expected relative risk by simulation can be made. The $P(1-P)$ in the previous equation denotes the variance of one observation. Since relative risk is bounded by 0 and 1, the maximum variance of any distribution of values between 0 and 1 is .25, the variance of the Bernoulli observation with probability .5. One can see this by noting that with that distribution, the distribution of the responses are as spead out as they could be, with half at 0 and half at 1.

A How to Use the `hbim` R package

This section details how to use the `hbim` R-package, the definitive software for the paper. A simplified version of the model is also available in an Excel version (see supplementary file "Combination Model Spreadsheet.zip").

A.1 Installation of R and `hbim` package

To use the package:

1. Download R (it is open source freeware, see `http://www.r-project.org/`) and install it. R runs on Windows, Linux or MacOS operating systems. In Windows, for example, there is an executable installation file. Use a version of R that is $\geq$ 2.5.0.

2. Run R and install the `hbim` package. The windows version of the package is available as the supplemental file `hbim_0.9.5.zip.` Versions for other operating systems (e.g., Linux) will be made available on the CRAN site (`http://www.r-project.org/`). Follow the help for your system. In Windows, save the zip file in a folder, use the drop down menus: packages>Install package(s) from local zip files.... and find the zip file in the folder where it was saved.

3. Install the `mvtnorm` package from CRAN (`http://www.r-project.org/`).

4. Load the package. At R prompt (denoted by >):

   >library(`hbim`); ?`hbim`

   This allows use of all functions in the package and calls up a help menu for each function.

To extract the code from the .Rnw file that produced this document, you need to find the file. After installation of the package, from the library subdirectory of your R directory (e.g., `C:\R\R-2.5.0\library`), the file is at `hbim \doc\inst\hbimdetails.Rnw`. Then from R type

`Stangle("put location of file here\hbimdetails.Rnw")`. For example

`Stangle("H:\\main\\malaria\\saul\\combination\\tex\\hbimdetails.Rnw")`. This will create a file called hbimdetails.R which will be put in your working R directory, which will have all the R code used to perform all the calculations of this document.

A.2 Access to Immune Response Database

After installing the `hbim` package one can access the immune response database. Here is the code to access the database and printout the 35th observation of the database:

```
> data(irdata)
> irdata[35, ]
```

```
   RecordNum Old.Reference Reference Vaccine.and.trial.group
35        35             6         5                GBS II-TT
   Carrier.for.conjugate.vaccines Age.in.yrs.at.first.vaccination
35                 Tetanus toxoid                           20-45
   Dose.schedule.in.weeks Num.Immunizations
35                      0                 1
   Endpoint.in.weeks.after.first.vaccine Antigen Units GMT
35                                     4  II-CPS    ug 6.7
   GMT.95.pct.interval.low.limit GMT.95.pct.interval.high.limit  n Fold.Range
35                           3.3                           13.5 25      993.8
```

R prints out a 35 at the beginning of each line to denote that all the lines are on the 35th row of the irdata. There are many variables, and some are missing values. For the calculations of this document we are

concerned with only a few variables.

The variable "Reference" gives the number of the reference associated with those data. For the 35th observation the reference number is 5. After installing the refs data (using `data(refs)`), we can get that reference by the command: refs[5]. The results will print:

```
Baker, C. J. et al. Safety and immunogenicity of a bivalent group B streptococcal conjugate vaccine for
50 Levels: Al Mazrou, Y. et al. Serologic responses to ACYW135 polysaccharide meningococcal vaccine in S
```

The first line is the reference. It is a long character string that does not fit on the format for this document. The second lines gives the list of all the references.

A.3 Calculation of Fold-Range from 95% Confidence Interval

The function used to calculate the fold-range from the confidence interval is `calc.foldrange`. Here is an example: suppose the 95% confidence interval for the mean antibody response is $(65.2, 87.6)$ from n =203 subjects. Then here is the R code to do that calculation, followed by the results:

```
> calc.foldrange(203, 65.2, 87.6)

    n lower upper     s.byt     s.byz foldrange.byt foldrange.byz
1 203  65.2  87.6 0.4633824 0.4661754      65.52782      67.20073
```

The value *s.byt* is s from equation 1, and *s.byz* is s from equation 2.

We can calculate the foldrange for the entire Immune Response database, and print out the first 5 results by the following:

```
> frall <- calc.foldrange(irdata$n, irdata$GMT.95.pct.interval.low.limit,
+     irdata$GMT.95.pct.interval.high.limit)
> frall[1:5, ]
```

```
    n lower upper     s.byt     s.byz foldrange.byt foldrange.byz
1 203  65.2  87.6 0.4633824 0.4661754      65.52782      67.20073
2 205  73.9  99.3 0.4658624 0.4686426      67.01114      68.71401
3 206  68.7  93.5 0.4872123 0.4901057      81.25256      83.40246
4 203 149.8 189.4 0.3680430 0.3702614      27.71419      28.27469
5 206 144.4 187.3 0.4111965 0.4136384      40.91294      41.82471
```

We can do statistics on the fold-ranges calculated from the data set. First note that there are 574 different data points from the data set. To explore the distribution of the fold-range values we look at some quantiles. Note the 0% quantile is the minimum, 100% quantile is the maximum, 50% quantile is the median, and 95% of the fold-range values are between the 2.5% quantile and the 97.5% quantile. Here are those values by both methods:

```
> quantile(frall[, "foldrange.byt"], probs = c(0, 0.025, 0.5, 0.975,
+     1))
          0%         2.5%           50%        97.5%          100%
    5.626696     9.130913     63.669538  4015.745669  68274.235562
> quantile(frall[, "foldrange.byz"], probs = c(0, 0.025, 0.5, 0.975,
+     1))
          0%         2.5%           50%        97.5%          100%
5.889425e+00 9.336993e+00 6.770472e+01 6.039323e+03 1.950395e+05
```

Also consider the quantiles of the standard deviation values.

```
> quantile(frall[, "s.byt"], probs = c(0, 0.025, 0.5, 0.975, 1))
       0%      2.5%       50%     97.5%      100%
0.1913947 0.2450334 0.4601947 0.9193417 1.2332514
```

```
> quantile(frall[, "s.byz"], probs = c(0, 0.025, 0.5, 0.975, 1))

       0%      2.5%       50%     97.5%      100%
0.1964508 0.2475038 0.4670032 0.9645071 1.3495459
```

Note that because each estimated fold range has some variability, these quantiles calculated from the estimates will produce a larger middle 95% range (i.e., smaller 2.5% quantile and larger 97.5% quantile) than the quantiles of the fold ranges that would result if it was possible to estimate each fold range without error. Because the only use of these quantiles is to posit a range of plausible values, we do not investigate the estimatation of the quantiles further.

A.4 Recreating Figure 1

We redo all the figures of the main paper in this document. In order to see them in the form of the paper (e.g., four panels on a page for Figures 3, 4, and 5), after loading the `hbim` package, type for example `demo("make.figure1")`.

The details of the creation of Figure 1 of the main paper are hidden in the .Rnw file in the fig1 code chunk. Briefly, only studies performed on infants with more than one study per antigen are presented. For completeness we recreate Figure 1 as Figure 1.

A.5 Recreating Figure 2

To create Figure 2 of the paper, we repeatedly calculate the integrals of equation 4 by numeric integration. For example, at the point 0.1 with two component variance model, with $\sigma = 0.462486395398815$, we assume that $\mu = [0.1, 0.1]$ and

$$\mathbf{V} = \begin{bmatrix} \sigma^2 & 0 \\ 0 & \sigma^2 \end{bmatrix}.$$

Here is the R code to calculate that. First note that :

```
> V <- matrix(c(sigmas[2], 0, 0, sigmas[2]), 2, 2)
> V
```

Figure 1: In the paper this is Figure 1.

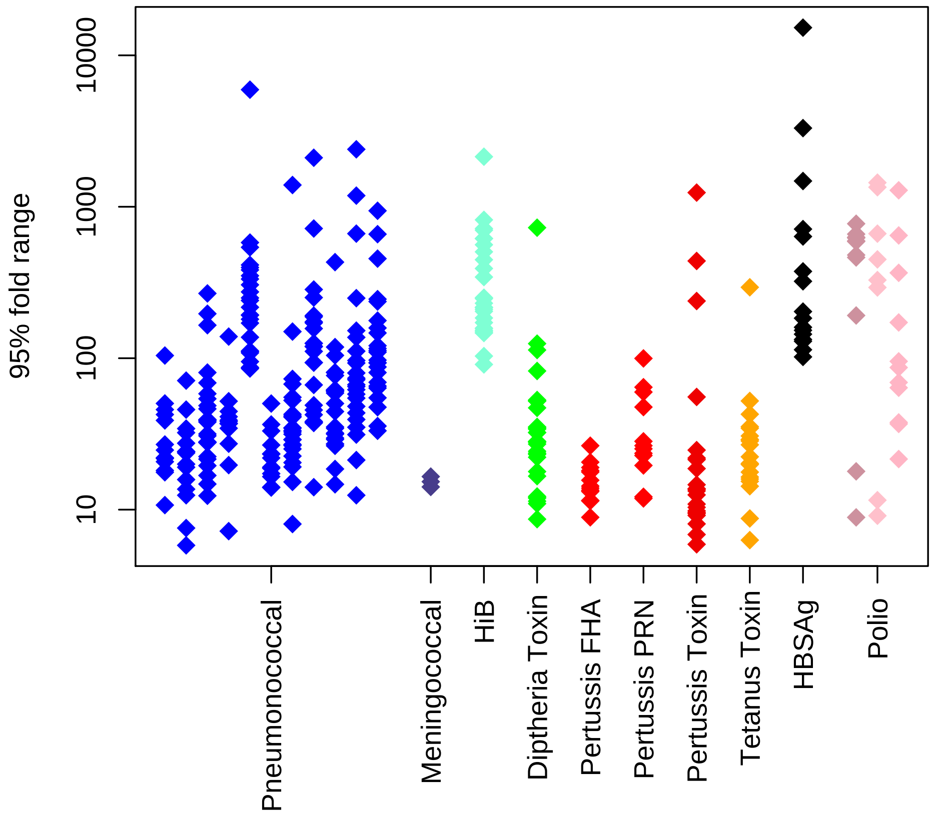

```
          [,1]      [,2]
[1,] 0.4624864 0.0000000
[2,] 0.0000000 0.4624864
```

```
> mu <- c(0.1, 0.1)
> hbrr(mu, V)
```

```
[1] 0.2117964
```

For Figure 2 we repeat these calculations at many points. We calculated the expected relative risk over different values of σ (see equation 4), where for each calculation of that equation we have let all the σ values be equal (i.e., $\sigma = \sigma_1 = \cdots = \sigma_3$). For different calculations of equation 4, the value of σ would change. The different values of σ used in the different calculations are determined in the PreliminaryCalculation code chunk, by the `SIGMAS` object. Specifically,

```
> SIGMAS
```

```
[1] 0.2434337 0.4624864 0.9436321
```

Since the creation of the data took several hours, we have saved it as a data set called `deff.sigma`. In R, one can do the plot by:

```
> data(deff.sigma)
> plotlogm.resp(deff.sigma)
```

In this document it is labeled as Figure 2.

A.6 Creating Figure 3a

In order to create Figure 3a, we use the function, `plotresp.mix` on the `deff.sigma` data created using the function `eff.sigma`.

We recreate that figure as Figure 3 of this document.

Figure 2: In the paper this is Figure 2. sigma= 0.243433684742747 is green,sigma= 0.462486395398815 is blue,sigma= 0.943632136486441 is red

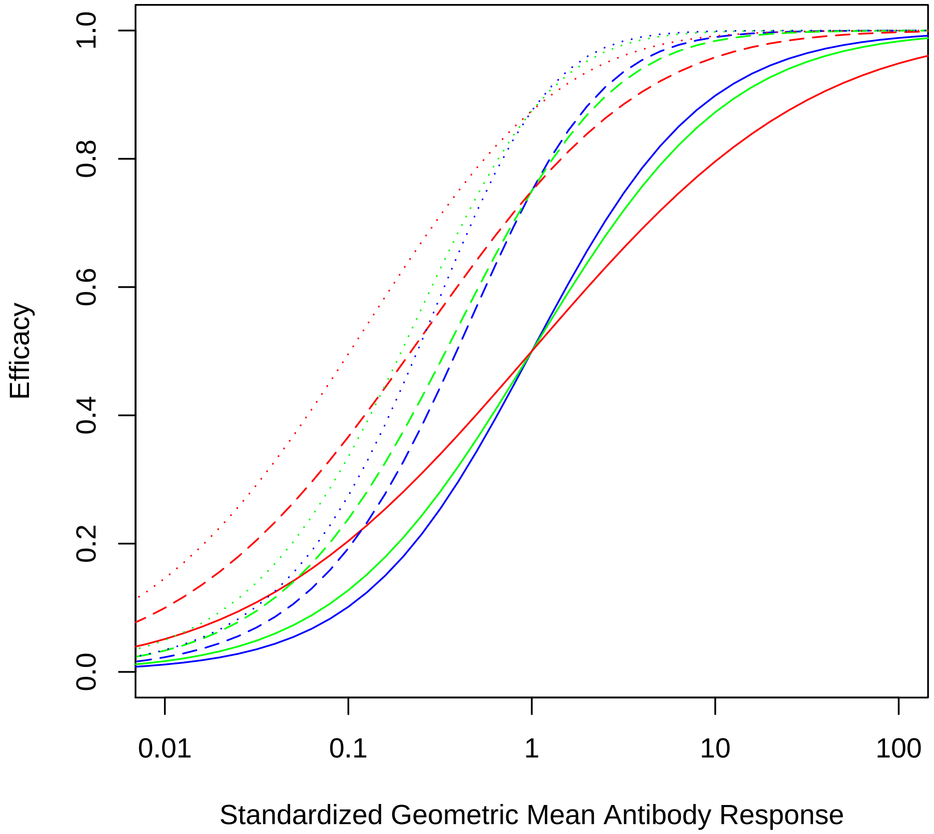

Figure 3: Figure 3a of Paper. sigma= 0.243433684742747 is green,sigma= 0.462486395398815 is blue,sigma= 0.943632136486441 is red

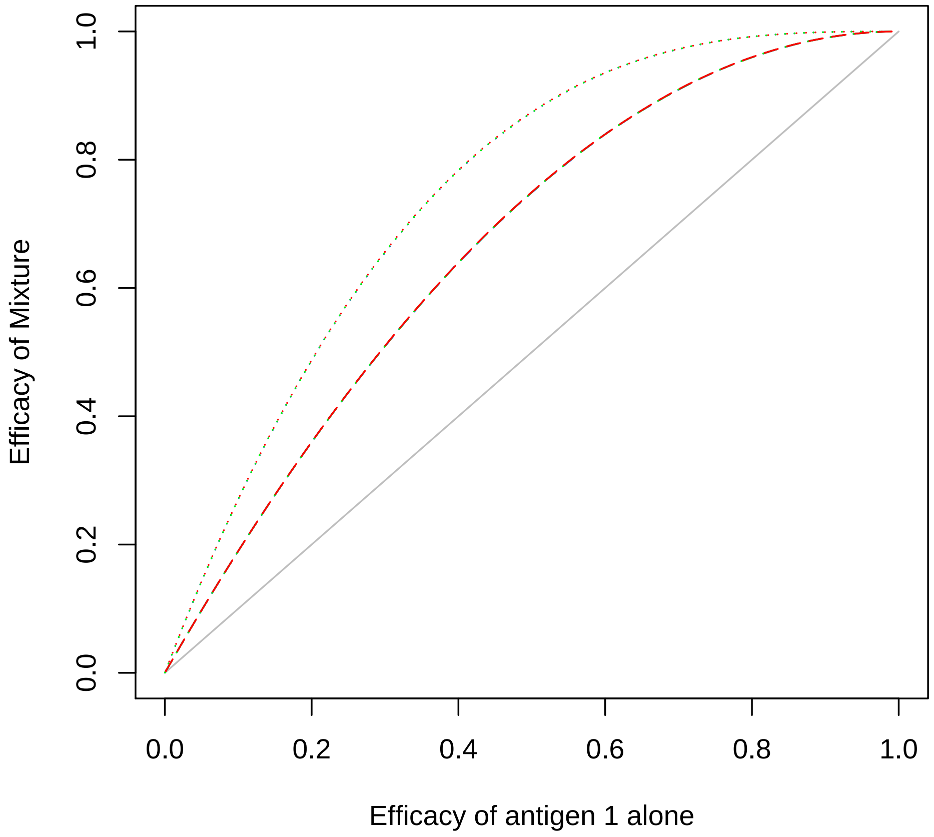

A.7 Creating Figure 3b

We now explain each point in Figure 3b. In Figure 4 of this document, we plot the lines from the single antigen and the two antigen combination from Figure 2 of the main paper evaluated at $\sigma = 0.243433684742747$. Consider an efficacy for a single antigen alone at e_1. There is an associated antigen level for a single antigen, a_1. Then if we had two antigens both at the level a_1 then the efficacy would be e_2. In order to get efficacy equal to e_2 with a single antigen, we need an amount of a_2 of a single antigen. Each point in the plot for Figure 3b from the paper is e_1 on the horizontal axis vs. a_2/a_1 on the vertical axis. The plot function uses the `equiv.increase` function. Here is the equivalent increase for the model with $\sigma_1 = \sigma_2 = SIGMAS[2] = 0.462486395398815$ for both the efficacy and the percent protected.

```
> D <- deff.sigma
> equiv.increase(D$mu, D$out1[, 2], D$mu, D$out2[, 2], 0.5)

$a1
[1] 4.295156e-15


$e2
[1] 0.75


$a2
[1] 0.5826686


$e1
[1] 0.5


$equiv.increase
```

```
[1] 3.825328

> D <- dpp.sigma

> equiv.increase(D$mu, D$out1[, 2], D$mu, D$out2[, 2], 50)

$a1

[1] 0.9542261


$e2

[1] 97.58574


$a2

[1] 1.872068


$e1

[1] 50


$equiv.increase

[1] 8.276407
```

We recreate Figure 3b as Figure 5 of this document.

A.8 Percent Protected and Associated Figures

In order to calculate percent protected we use the function, we use the function `pp.sigma` (see help for that function). Since the creation of the data took several hours, we have saved it as a data set called `dpp.sigma`. To have acces to that data use `data(dpp.sigma)`. Although not included in the main paper we can plot the percent protected using the `plotlogm.resp` function as:

Figure 4: Explanatory Plot for Figure 3b of Paper.

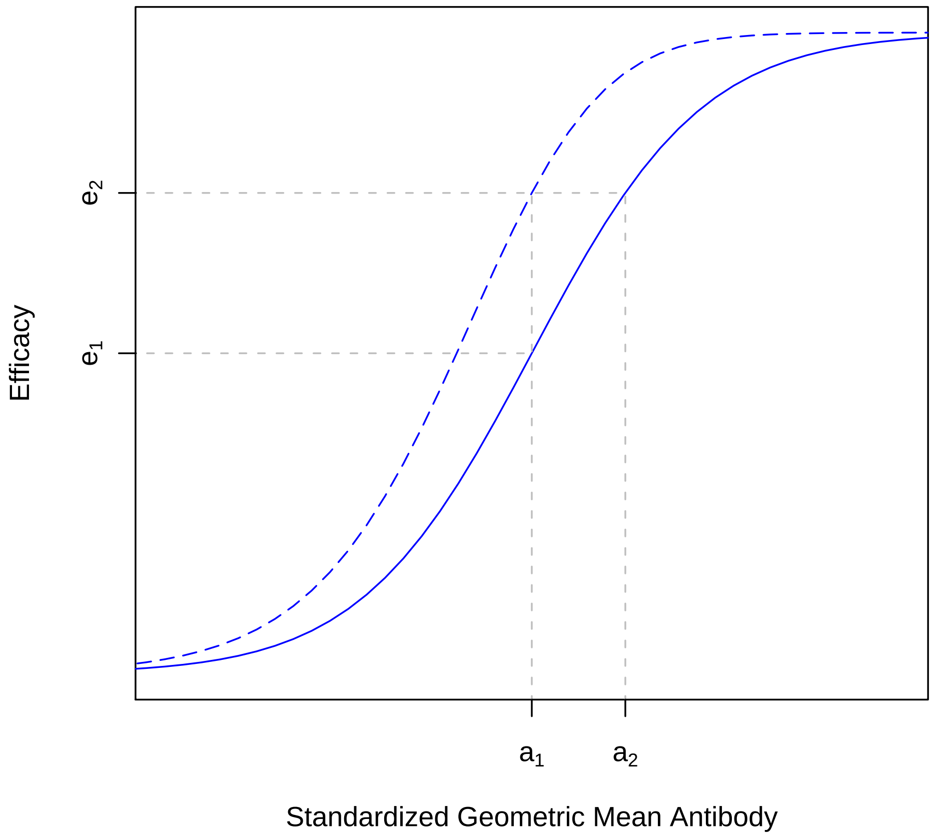

Figure 5: Figure 3b of Paper. sigma= 0.243433684742747 is green,sigma= 0.462486395398815 is blue,sigma= 0.943632136486441 is red

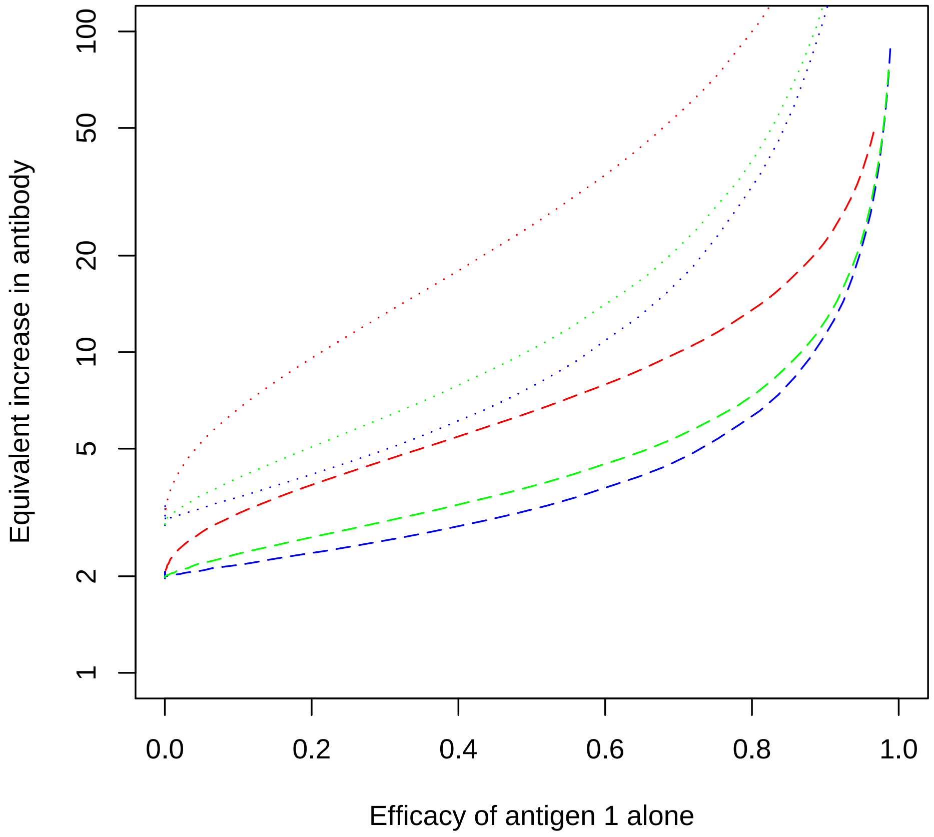

Figure 6: Standardized Geometric Mean Antibody by Percent Protected. sigma= 0.243433684742747 is green,sigma= 0.462486395398815 is blue,sigma= 0.943632136486441 is red

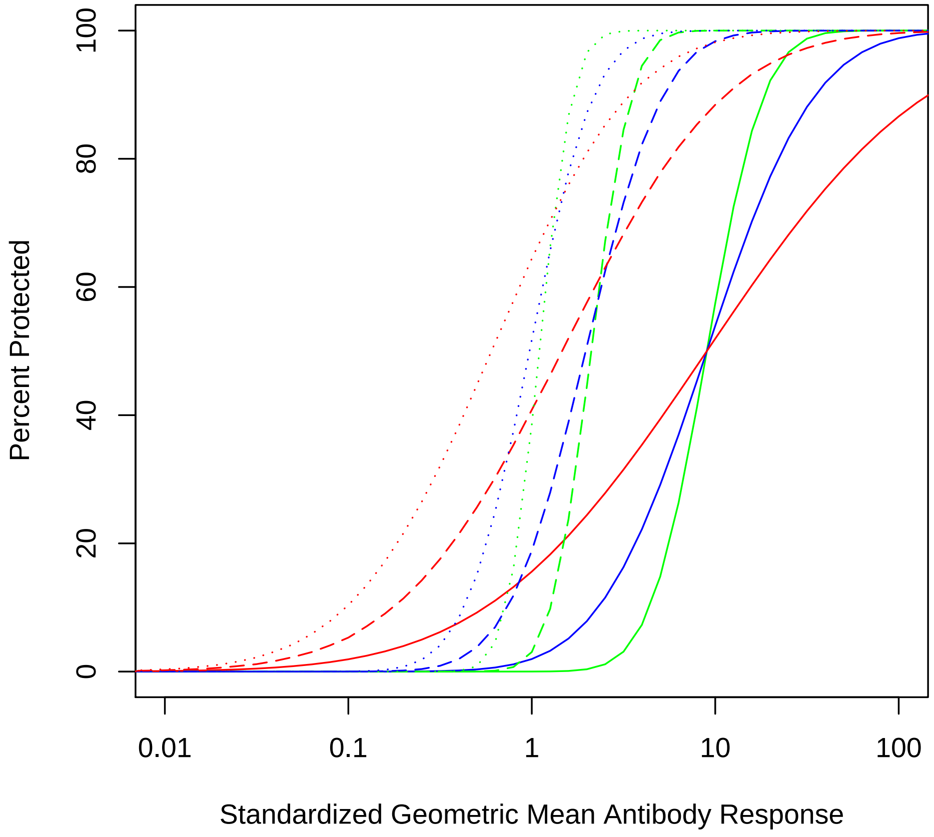


```
plotlogm.resp(dpp.sigma,YLIM=c(0,100),YLAB="Percent Protected")
```

which is plotted as Figure 6.

To create Figure 3c of the main paper, we use:

```
plotresp.mix(dpp.sigma,XYLIM=c(0,100),RLAB="% Protected by").
```

It is Figure 7 of this document.

To create Figure 3d of the main paper, there are some issues. First recall how Figure 3b of the main paper was calculated (Figure 5 of this document). Compare Figure 4 and 6 of this document. One can see

Figure 7: Figure 3c of Paper. sigma= 0.243433684742747 is green,sigma= 0.462486395398815 is blue,sigma= 0.943632136486441 is red

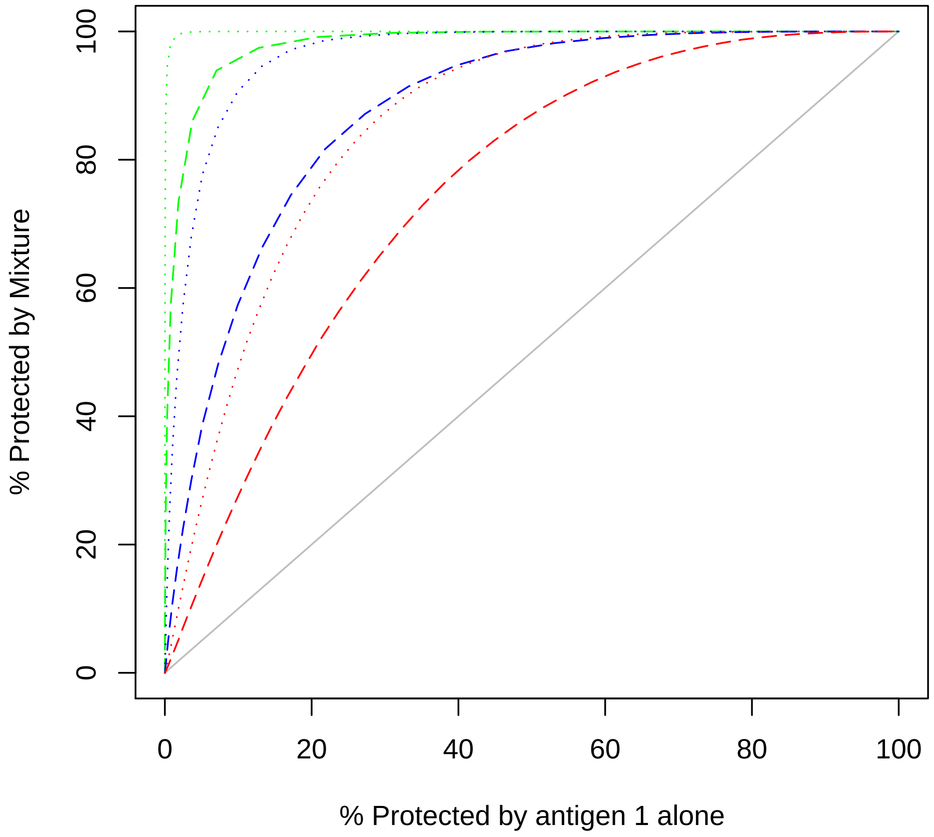

Figure 8: First Try at Figure 3d of Paper. sigma= 0.243433684742747 is green,sigma= 0.462486395398815 is blue,sigma= 0.943632136486441 is red

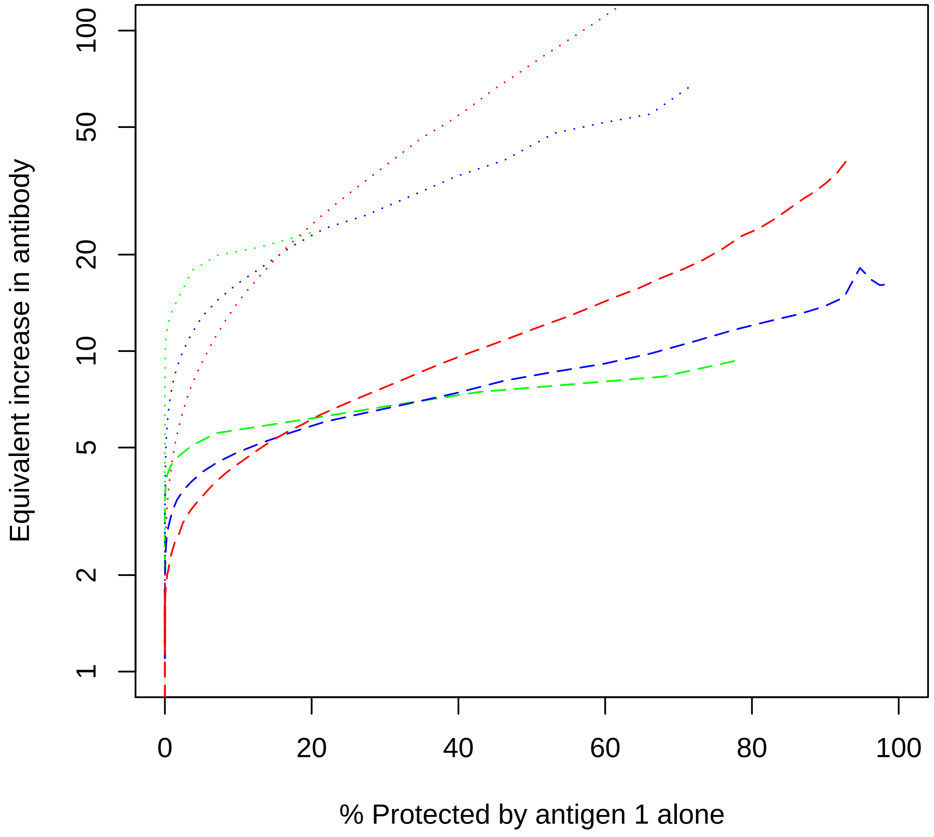

how many of the points will be very hard to estimate because the dose response curves become essentially flat at low and high percent protected values. Figure 8 of this document is a first try and Figure 3d of the main paper.

We do not print the values which are very hard to estimate. Return to Figure 4. In this case, e_1 and e_2 represent percent protection. We limit the lines to cases where $0.01 < e_2 < 99.9$. This is how we create Figure 3d of the main paper; it is Figure 9 of this document. We use those same limits for Figures 4d and 5d of the main paper (i.e., Figures 15 and 21 of this document).

Figure 9: Figure 3d of Paper. sigma= 0.243433684742747 is green,sigma= 0.462486395398815 is blue,sigma= 0.943632136486441 is red

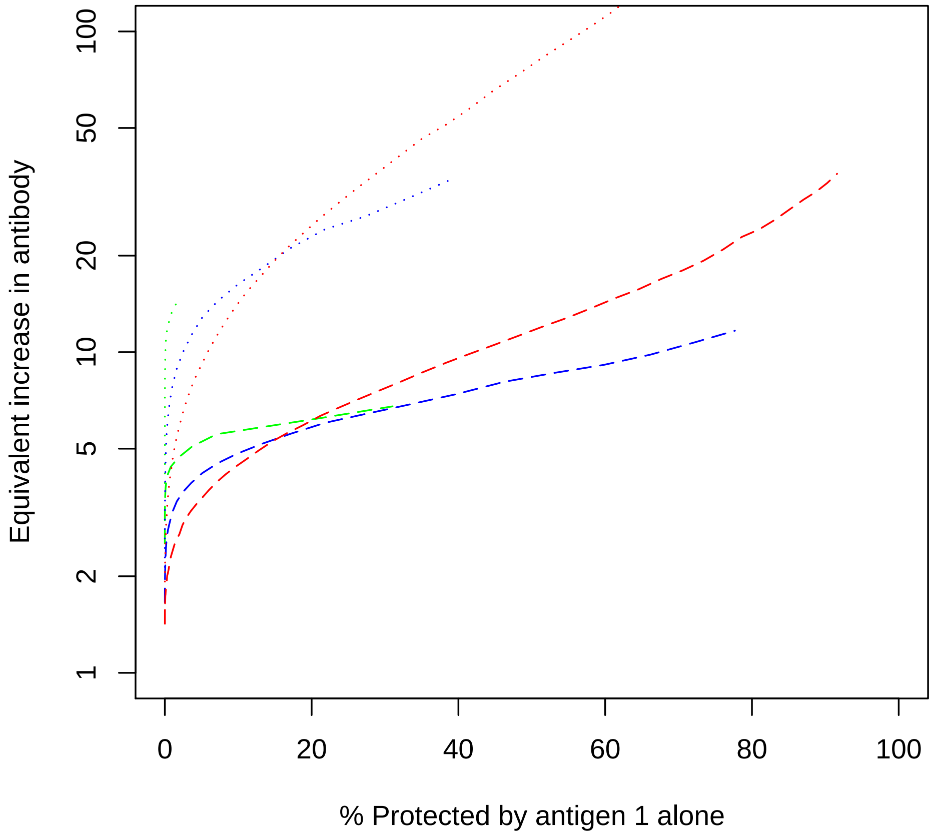

A.9 Creating Figures 4a,b,c and d

In order to create Figures 4 of the main paper we created data using the function `eff.mu` and `pp.mu` (see help for those functions). Essentially, we calculated the expected relative risk over different values of μ (see equation 4). For the single component vaccine we use the μ value that can be read from the plots as 10^{μ}. For the second and third component of the vaccine we set $\mu_2 = \mu_3 = \mu_1 + \log 10(FACTOR)$, where FACTOR is some factor (1/2, 1/3, etc.). In the plots, the median antibody plotted is 10^{μ_1}. We used these same values of μ_1 and μ_2 to calculate the percent protected. The different values of that factor used in the different calculations are determined in the PreliminaryCalculation code chunk, by the `FACTORS` object. Specifically,

```
> FACTORS

[1] 0.1000000 0.3333333 0.5000000 1.0000000
```

We create the figures similarly to Figures 3 of the main paper (see above). In Figures 10 and 11 we create figures similar to Figure 2 of the main paper, except with the `deff.mu` and `dpp.mu` output. The Figures 4a, 4b, 4c, and 4d of the main paper are labeled Figures 12, 13, 14, and 15 in this document.

A.10 Creating Figures 5a,b,c and d

In order to create Figures 5 of the main paper we created data using the function `eff.rho` and `pp.rho` (see help for those functions). Essentially, we calculated the expected relative risk over different values of ρ (see equation 4). For the single component vaccine there is no ρ parameter, but there is one in the two and three component vaccines. The different values of ρ used in the different calculations are determined in the PreliminaryCalculation code chunk, by the `RHOS` object. Specifically,

```
> RHOS

[1] -0.50 -0.25  0.00  0.25  0.50  0.75  1.00
```

There were some integration problems so we calculated the expected efficacy values by simulation using 5e+05 simulations. Since efficacy is bounded by 0 and 1, similar (but not equivalent) statements about the

Figure 10: Efficacy when Changing Means by Factors. factor= 0.1 is red,factor= 0.333333333333333 is green,factor= 0.5 is blue,factor= 1 is black

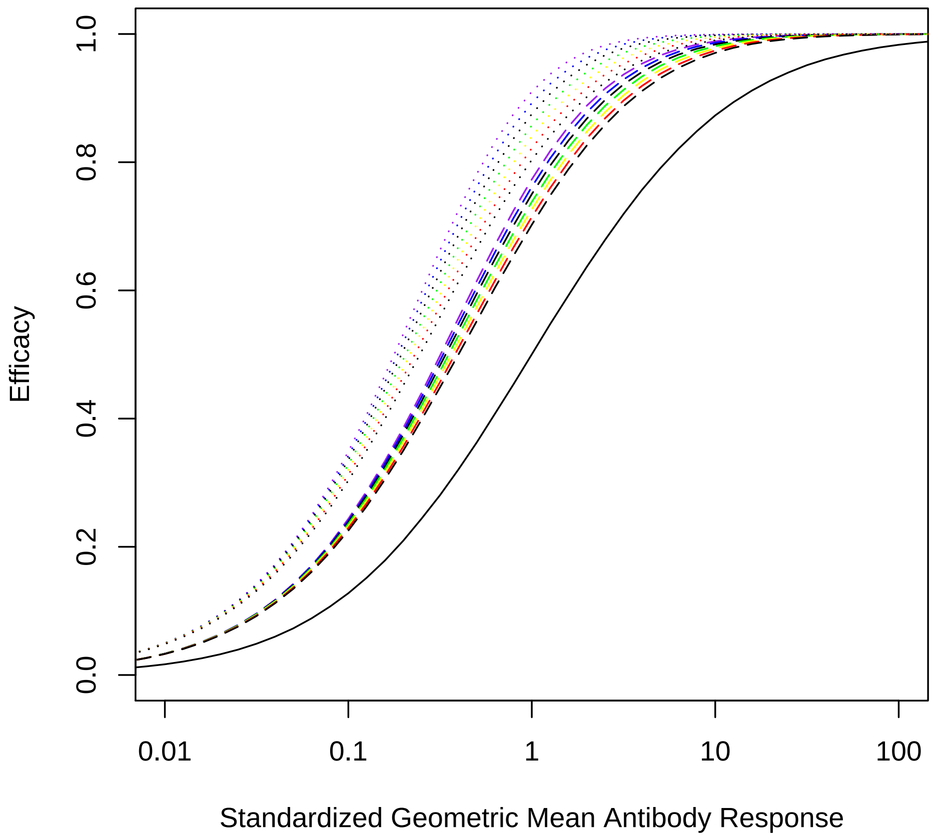

Figure 11: Percent Protected when Changing Means by Factors. factor= 0.1 is red,factor= 0.333333333333333 is green,factor= 0.5 is blue,factor= 1 is black

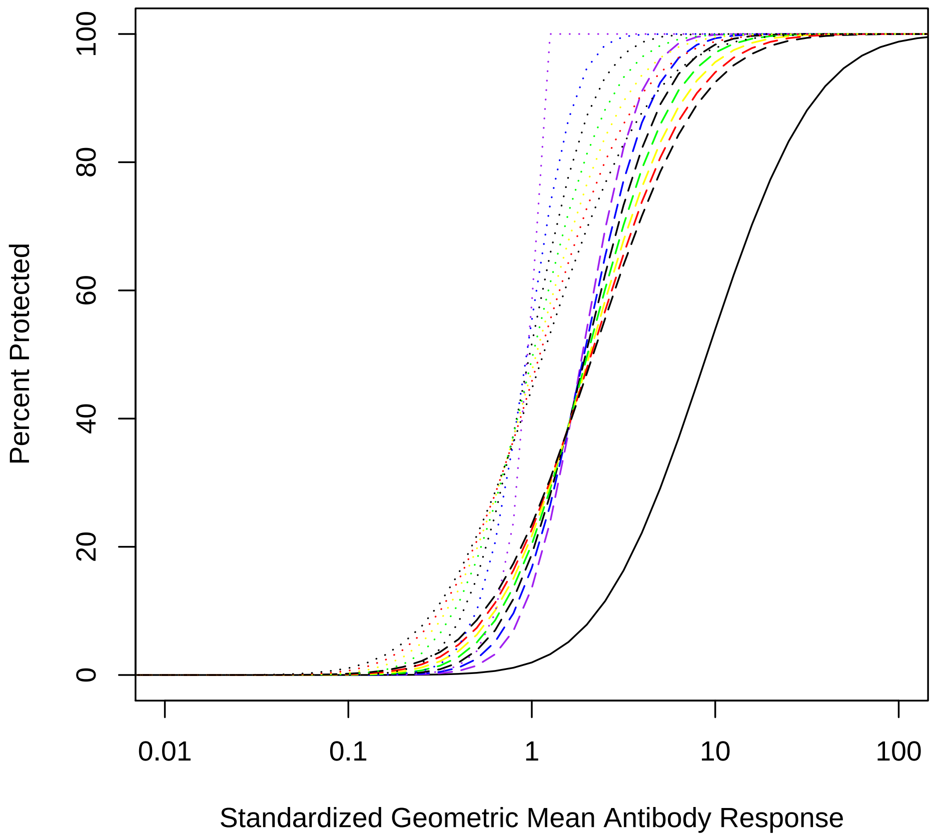

Figure 12: Figure 4a of Paper. factor= 0.1 is red,factor= 0.333333333333333 is green,factor= 0.5 is blue,factor= 1 is black

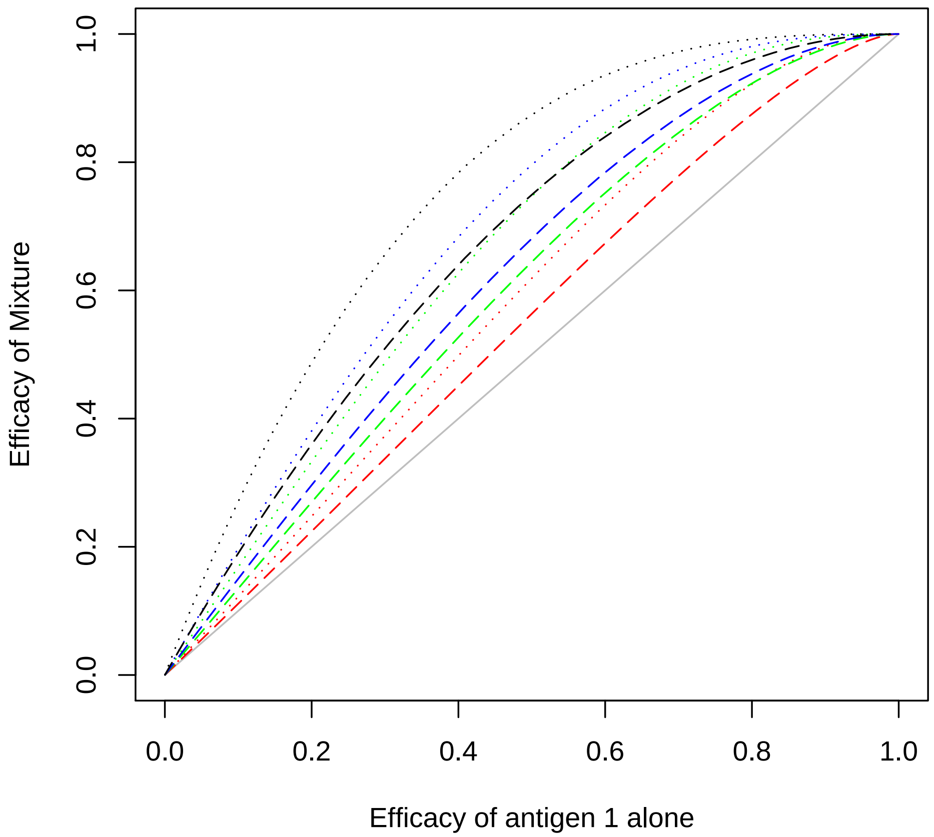

Figure 13: Figure 4b of Paper. factor= 0.1 is red,factor= 0.333333333333333 is green,factor= 0.5 is blue,factor= 1 is black

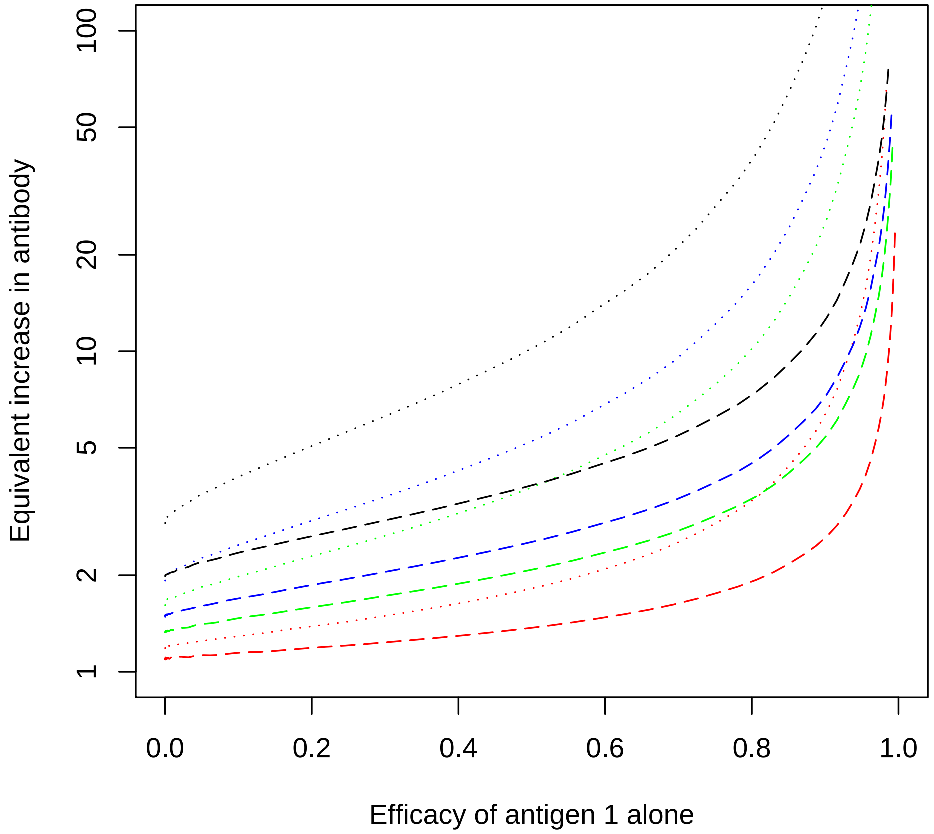

Figure 14: Figure 4c of Paper. factor= 0.1 is red,factor= 0.333333333333333 is green,factor= 0.5 is blue,factor= 1 is black

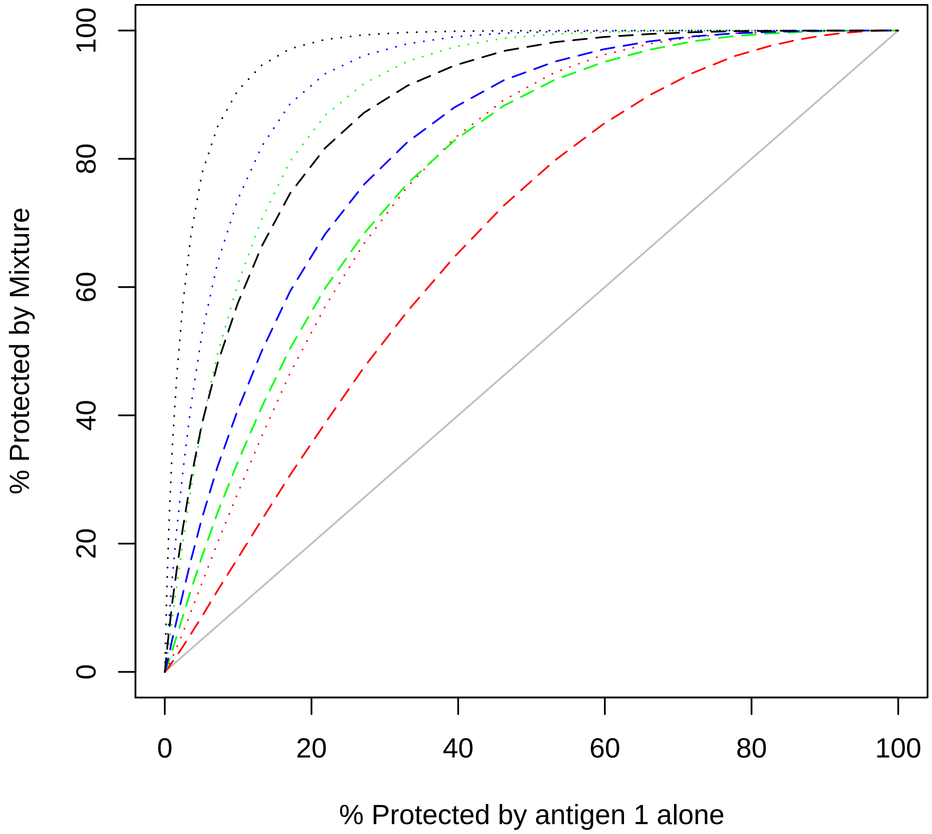

Figure 15: Figure 4d of Paper. factor= 0.1 is red,factor= 0.333333333333333 is green,factor= 0.5 is blue,factor= 1 is black

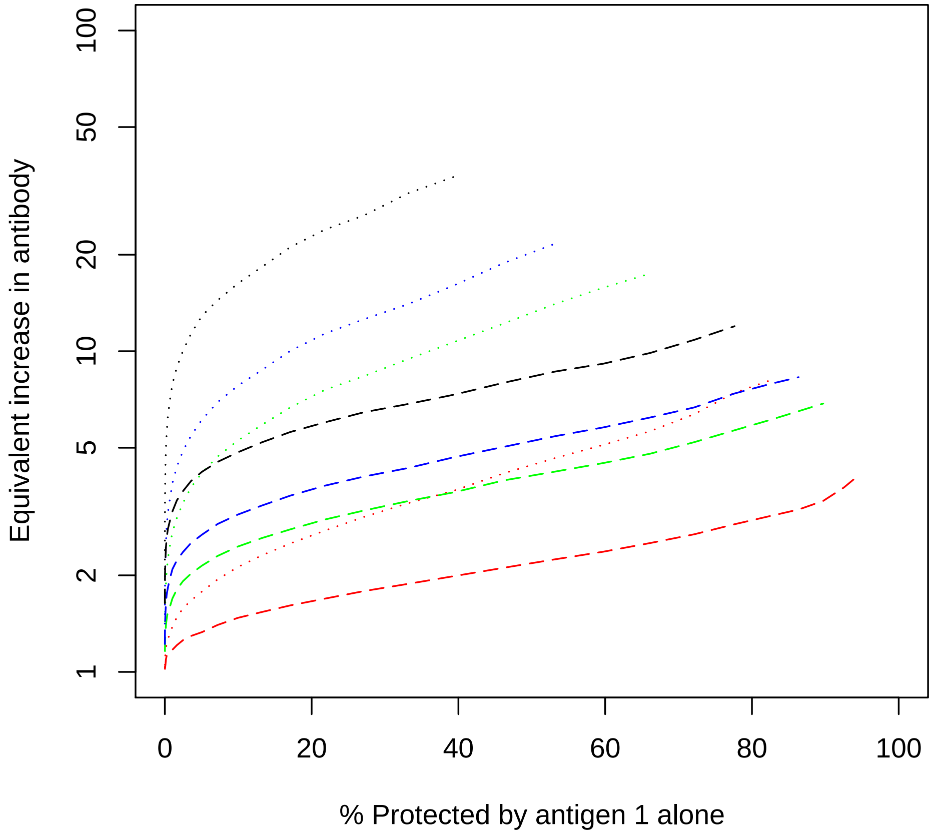

Figure 16: Efficacy when Changing Rho. rho= -0.5 is purple,rho= -0.25 is blue,rho= 0 is black,rho= 0.25 is green,rho= 0.5 is yellow,rho= 0.75 is red,rho= 1 is black

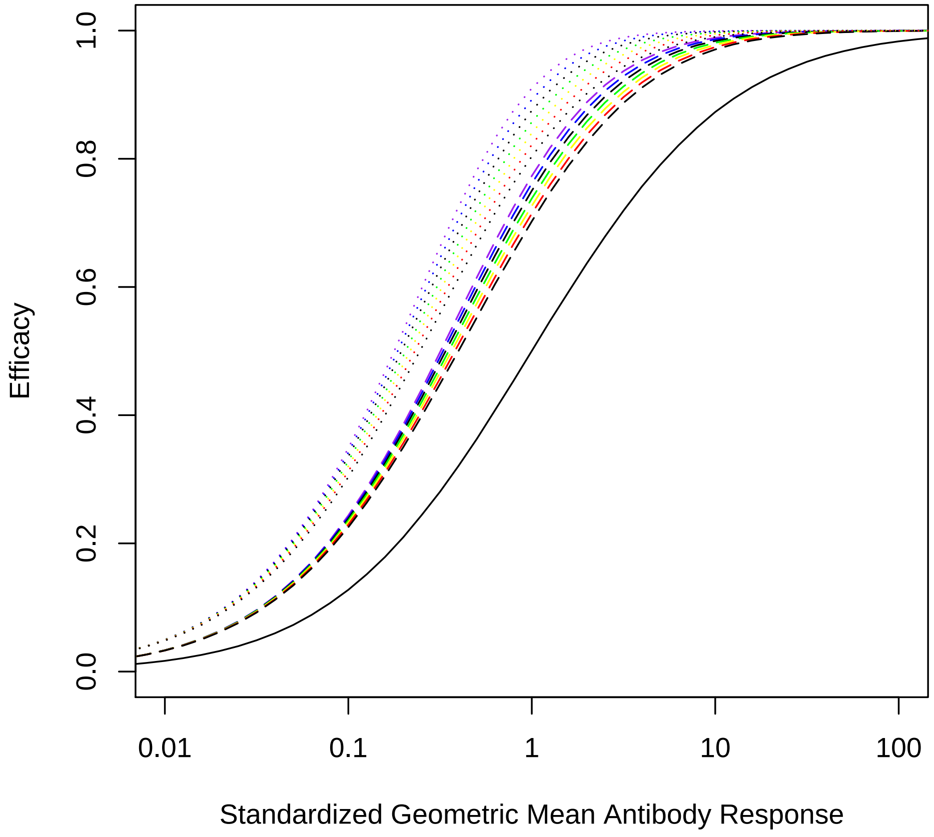

accuracy of efficacy can be made about these simulations as were made about simulation of the percent protected in Section 5.

We create the figures similarly to Figures 3 of the main paper (see above). In Figures 16 and 17 we create figures similar to Figure 2 of the main paper, except with the `deff.rho` and `dpp.rho` output. The Figures 5a, 5b, 5c, and 5d of the main paper are labeled Figures 18, 19, 20, and 21 in this document.

Figure 17: Percent Protected when Changing Rho. rho= -0.5 is purple,rho= -0.25 is blue,rho= 0 is black,rho= 0.25 is green,rho= 0.5 is yellow,rho= 0.75 is red,rho= 1 is black

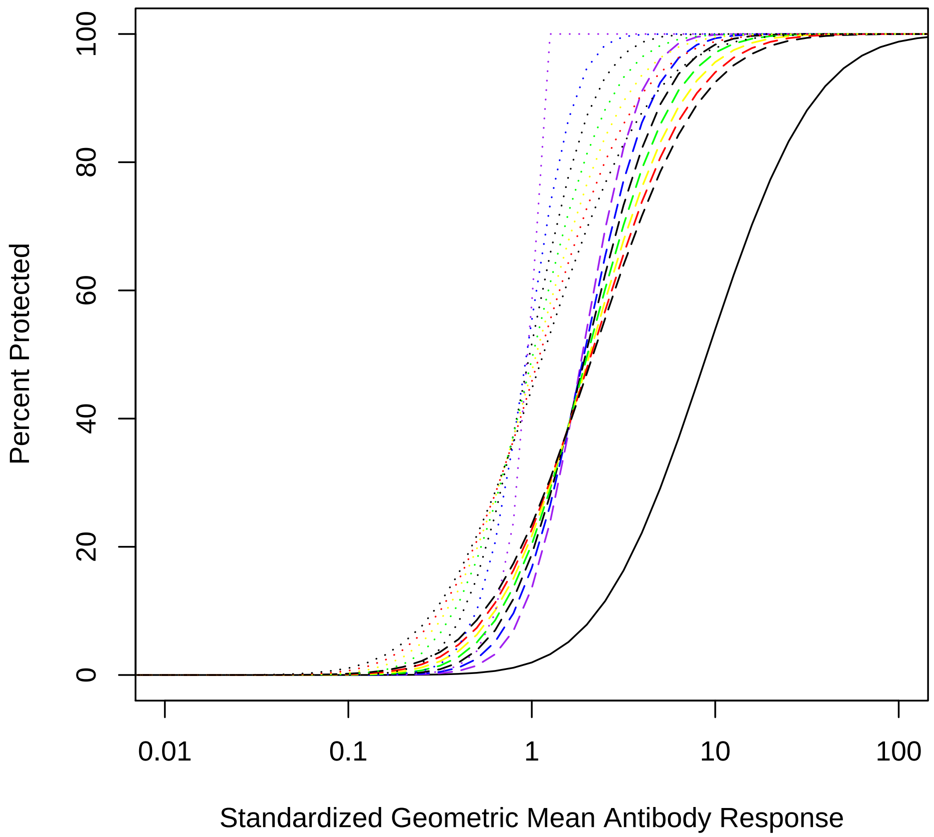

Figure 18: Figure 5a of Paper. rho= -0.5 is purple,rho= -0.25 is blue,rho= 0 is black,rho= 0.25 is green,rho= 0.5 is yellow,rho= 0.75 is red,rho= 1 is black

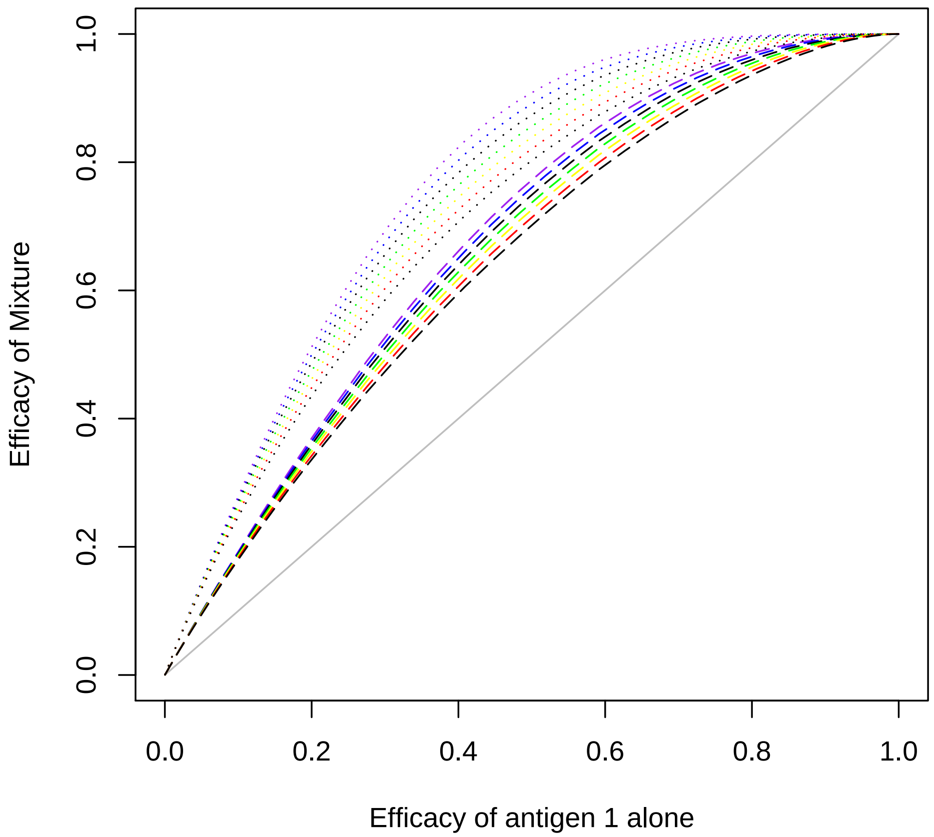

Figure 19: Figure 5b of Paper. rho= -0.5 is purple,rho= -0.25 is blue,rho= 0 is black,rho= 0.25 is green,rho= 0.5 is yellow,rho= 0.75 is red,rho= 1 is black

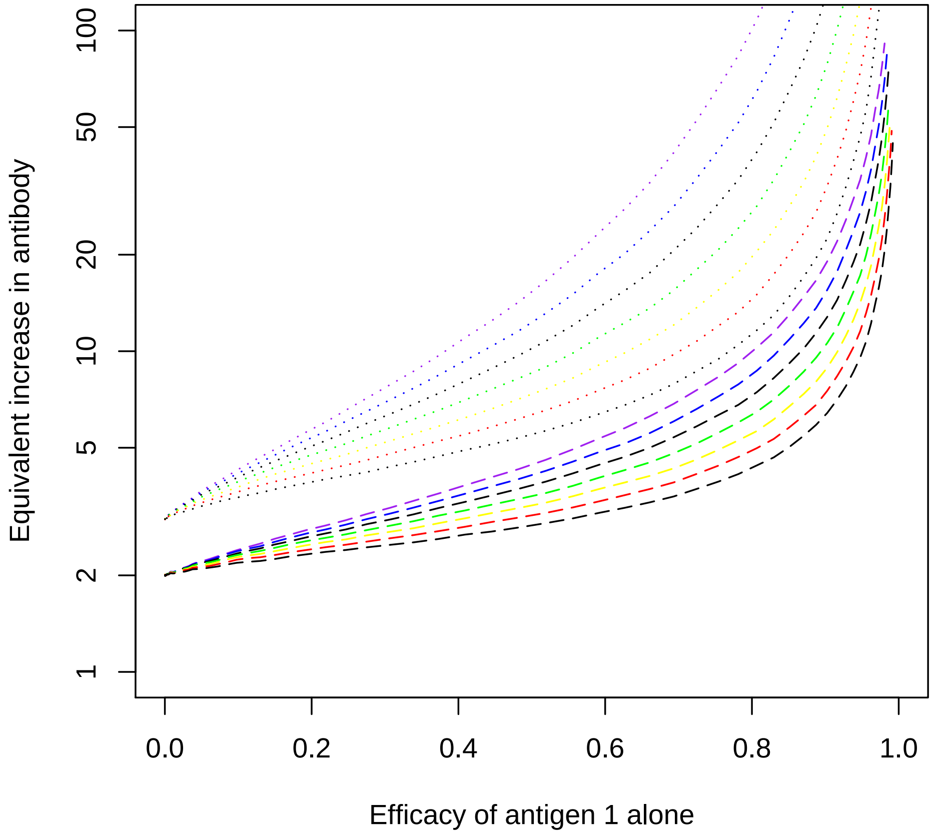

Figure 20: Figure 5c of Paper. rho= -0.5 is purple,rho= -0.25 is blue,rho= 0 is black,rho= 0.25 is green,rho= 0.5 is yellow,rho= 0.75 is red,rho= 1 is black

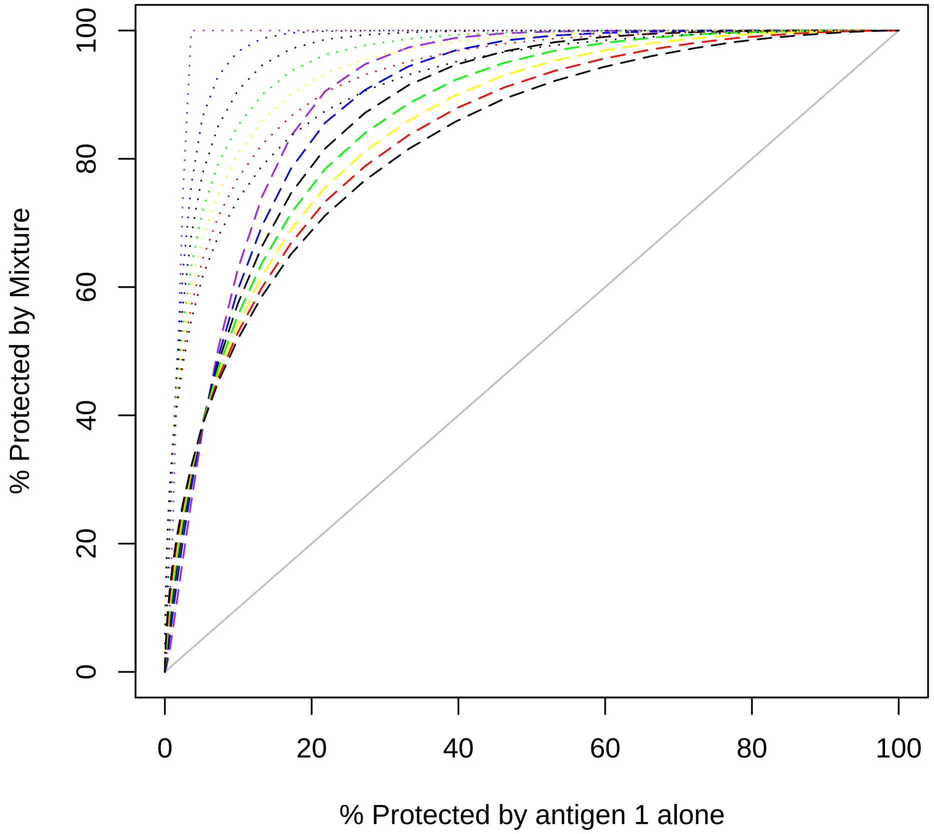

Figure 21: Figure 5d of Paper. rho= -0.5 is purple,rho= -0.25 is blue,rho= 0 is black,rho= 0.25 is green,rho= 0.5 is yellow,rho= 0.75 is red,rho= 1 is black

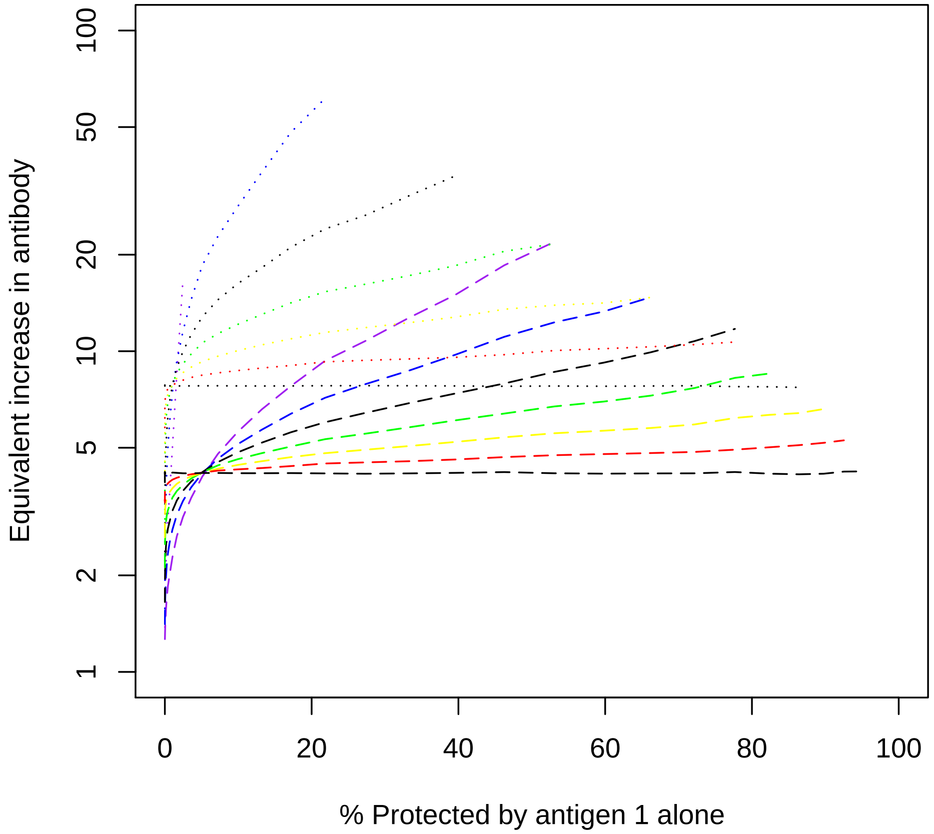

B Lognormal Distribution

Here are some properties of the lognormal distribution (see e.g., Antle, 1985):

Suppose that $Y = \log_e(X)$ has a normal distribution with mean equal μ and variance equal σ^2. Then X has a lognormal distribution with mean equal to

- $E(X) = \exp\left(\mu + \frac{\sigma^2}{2}\right)$ and
- $Var(X) = \exp\left(2\mu + \sigma^2\right)\left\{\exp(\sigma^2) - 1\right\}$

This means that the coefficient of variation of X (denoted $CV(X)$) is

$$CV(X) = \frac{\sqrt{Var(X)}}{E(X)} = \frac{\sqrt{\exp(2\mu + \sigma^2 \left\{\exp(\sigma^2) - 1\right\}}}{\exp\left(\mu + \frac{\sigma^2}{2}\right)} = \sqrt{\exp(\sigma^2) - 1}$$

For the lognormal distribution σ is sometimes known as the shape parameter. If we let c =coefficient of variation, then

$$\sigma^2 = \log_e(c^2 + 1)$$

Now suppose that $Z = \log_{10}(X) = Y \log_{10}(e)$. Then Z has normal distribution with mean equal to $\mu \log_{10}(e)$ and variance equal to $\log_{10}(2e)\sigma^2$ and the coefficient of variation is the same as above. This is like a rescaling of the units on the log scale. We can go through a change to those units if we want; it does not change the basic relationships between means and variances.

References

Antle, C.E. (1985) "Lognormal Distribution" in *Enclyclopedia of Statistics* Vol. 5. (editors: S. Kotz and N.L. Johnson) , Wiley: New York.

Casella, G., and Berger, R.L. (2002). *Statistical Inference, second edition* Duxbury: Pacific Grove, CA.

Gentleman, R. and Temple Lang, D. (2007). "Statistical Analyses and Reproducible Research". Journal of Computational and Graphical Statistics, **16**: 1-23. Earlier version available at: `http://www.bepress.com/bioconductor/paper2`.

Gentleman, R. (2005) "Reproducible Research: A Bioinformatics Case Study," Statistical Applications in Genetics and Molecular Biology: Vol. 4 : Iss. 1, Article 2. Available at: `http://www.bepress.com/sagmb/vol4/iss1/art2`

Greco, W.R., Bravo, G., and Parsons, J.C. (1995). "The search for synergy: a critical review from a response surface perspective" *Pharmacological Reviews* **47**, 331-385.

R Development Core Team (2007). R: A language and environment for statistical computing. R Foundation for Statistical Computing, Vienna, Austria. ISBN 3-900051-07-0, URL http://www.R-project.org.
