# Supplementary material for: Human Immunity and the Design of Multi-Component, Single Target Vaccines
Source: PLoS One. 2007 Sep 5;2(9):e850. doi: 10.1371/journal.pone.0000850 (PMC1952173; doi:10.1371/journal.pone.0000850)
Supplement: Software S1 — Multi-component, single target vaccine R program software package. The R package containing the model. Instructions for unzipping and installing this program are contained in the supplementary file Hbimdetails.pdf (0.60 MB ZIP) [file pone.0000850.s004.zip › hbim/html/00Index.html]

R: Hill/Bliss Independence Model for Combination Vaccines

# Hill/Bliss Independence Model for Combination Vaccines

---

## Documentation for package `hbim' version 0.9.5

## User Guides and Package Vignettes

Read overview or browse directory.

## Help Pages

|  |  |
| --- | --- |
| hbim-package | Hill/Bliss Independence Model for Multicomponent Vaccines |
| calc.foldrange | Calculate stadard error and fold-range from confidence interval |
| deff.mu | HBIM data |
| deff.rho | HBIM data |
| deff.sigma | HBIM data |
| dpp.mu | HBIM data |
| dpp.rho | HBIM data |
| dpp.sigma | HBIM data |
| eff.mu | Create data sets for plots |
| eff.rho | Create data sets for plots |
| eff.sigma | Create data sets for plots |
| equiv.ab | Equivalent antibody calculations by Linear Interpolation |
| equiv.increase | Calculate equivalent increase from two dose-response curves |
| hbim | Hill/Bliss Independence Model for Multicomponent Vaccines |
| hbpp | Calculate expected relative risk or percent protected from Hill model with Bliss Independence |
| hbrr | Calculate expected relative risk or percent protected from Hill model with Bliss Independence |
| irdata | Immune Response data |
| make.v | Make Exchangeable Variance Matrix |
| plotlogm.resp | Plot Hill/Bliss Independence Model Data. |
| plotresp.equiv | Plot Hill/Bliss Independence Model Data. |
| plotresp.mix | Plot Hill/Bliss Independence Model Data. |
| pp.mu | Create data sets for plots |
| pp.rho | Create data sets for plots |
| pp.sigma | Create data sets for plots |
| refs | Reference list |
