# Supplementary material for: Human Immunity and the Design of Multi-Component, Single Target Vaccines
Source: PLoS One. 2007 Sep 5;2(9):e850. doi: 10.1371/journal.pone.0000850 (PMC1952173; doi:10.1371/journal.pone.0000850)
Supplement: Software S1 — Multi-component, single target vaccine R program software package. The R package containing the model. Instructions for unzipping and installing this program are contained in the supplementary file Hbimdetails.pdf (0.60 MB ZIP) [file pone.0000850.s004.zip › hbim/html/calc.foldrange.html]

R: Calculate stadard error and fold-range from confidence interval

|  |  |
| --- | --- |
| calc.foldrange {hbim} | R Documentation |

## Calculate stadard error and fold-range from confidence interval

### Description

Given a confidence interval and sample size, we find the standard error assuming confidence limits
are calculated on the log10 responses by either normal confidence limits or t-distribution confidence limits.
The fold-range is also output by either methods.

### Usage

```
calc.foldrange(n, lower, upper, conf.level = 0.95)
```

### Arguments

|  |  |
| --- | --- |
| `n` | vector of sample size(s) used to create confidence intervals |
| `lower` | vector of lower confidence limits |
| `upper` | vector of upper confidence limits |
| `conf.level` | confidence level, default=.95 |

### Details

See `vignette("hbimdetails")`

### Value

A vector (or matrix) with elements (or columns)

|  |  |
| --- | --- |
| `n` | sample size |
| `lower` | lower confidence limit |
| `upper` | upper confidence limit |
| `s.byt` | standard deviation assuming confidence intervals calculated by t-distribution |
| `s.byz` | standard deviation assuming confidence intervals calculated by normal distribution |
| `foldrange.byt` | fold-range assuming confidence intervals calculated by t-distribution |
| `foldrange.byz` | fold-range assuming confidence intervals calculated by normal distribution |

### Examples

```
## sample size=43, lower cl=65, upper cl=85
calc.foldrange(43,65,85)
```

---

[Package *hbim* version 0.9.5 Index]
