# Supplementary material for: Human Immunity and the Design of Multi-Component, Single Target Vaccines
Source: PLoS One. 2007 Sep 5;2(9):e850. doi: 10.1371/journal.pone.0000850 (PMC1952173; doi:10.1371/journal.pone.0000850)
Supplement: Software S1 — Multi-component, single target vaccine R program software package. The R package containing the model. Instructions for unzipping and installing this program are contained in the supplementary file Hbimdetails.pdf (0.60 MB ZIP) [file pone.0000850.s004.zip › hbim/html/deff.sigma.html]

R: HBIM data

|  |  |
| --- | --- |
| deff.sigma {hbim} | R Documentation |

## HBIM data

### Description

These 6 data sets were calculated using the associated function. For example, `deff.sigma` was calculated with
`eff.sigma`. The 3 data sets that begin with deff, give the expected efficacy for several values of mu.
The 3 data sets that begin with dpp give the percent protected with several values of mu. The data sets that end in .sigma change
for different values of sigma, and similarly for .mu and .rho (see `deff.sigma`).

### Usage

```
data(deff.sigma)
data(deff.mu)
data(deff.rho)
data(dpp.sigma)
data(dpp.mu)
data(dpp.rho)
```

### Format

The format is:
List of 8

mu
:   vector of different values of mean for log10 antibody

out1
:   matrix of either expected efficacy or percent protected for 1 component model, rows corespond to mu, cols correspond to cparms

out2
:   matrix of either expected efficacy or percent protected for 2 component model, rows corespond to mu, cols correspond to cparms

out3
:   matrix of either expected efficacy or percent protected for 3 component model, rows corespond to mu, cols correspond to cparms

col1
:   vector of colors for different cparms of 1 component model

col2
:   vector of colors for different cparms of 2 component model

col3
:   vector of colors for different cparms of 3 component model

cparms
:   vector parameters that change

### Examples

```
## here is the code that produces the 6 data sets, it takes about 25 hours to run
## so it is commented out here
#NSIM<-5*10^5
#SIGMAS.POWER<-c(9,65,5000)
#SIGMAS<-log10(SIGMAS.POWER)/(2*qnorm(.975))
#SCOLORS<-c("green","blue","red")
#FACTORS<-c(1/10, 1/3, 1/2, 1)
#FCOLORS<-c("red", "green", "blue", "black")
#RHOS<-c(-.5,-.25,0, 0.25, 0.5, 0.75, 1)
#RCOLORS<- c("black","tan","yellow","blue", "green", "red", "black")
#set.seed(1234521)
#MU<-((-40:40)/10)
#deff.sigma<-eff.sigma(mu=MU, sigmas=SIGMAS, COLORS = SCOLORS, rho = 0)
#deff.mu<-eff.mu(mu=MU, factor = FACTORS, COLORS = FCOLORS, sigma = SIGMAS[2], rho = 0)
#deff.rho<-eff.rho(mu=MU, sigma = SIGMAS[2], rho = RHOS, COLORS =RCOLORS,simulate=TRUE,nsim=NSIM)
#set.seed(32401)
#dpp.sigma<-pp.sigma(MU,sigmas=SIGMAS,COLORS = SCOLORS, rho = 0,nsim=NSIM)
#set.seed(21345123)
#dpp.mu<-pp.mu(MU,factor = FACTORS, COLORS = FCOLORS, sigma = SIGMAS[2], rho = 0, nsim=NSIM)
#set.seed(435919)
#dpp.rho<-pp.rho(MU,sigma = SIGMAS[2], rho = RHOS, COLORS =RCOLORS,nsim=NSIM)
```

---

[Package *hbim* version 0.9.5 Index]
