# Supplementary material for: Human Immunity and the Design of Multi-Component, Single Target Vaccines
Source: PLoS One. 2007 Sep 5;2(9):e850. doi: 10.1371/journal.pone.0000850 (PMC1952173; doi:10.1371/journal.pone.0000850)
Supplement: Software S1 — Multi-component, single target vaccine R program software package. The R package containing the model. Instructions for unzipping and installing this program are contained in the supplementary file Hbimdetails.pdf (0.60 MB ZIP) [file pone.0000850.s004.zip › hbim/html/eff.mu.html]

R: Create data sets for plots

|  |  |
| --- | --- |
| eff.mu {hbim} | R Documentation |

## Create data sets for plots

### Description

These functions create the data sets used in the plots. The first part of the name denotes the
output created. Thus, `eff.sigma`, `eff.mu`, `eff.rho` create efficacy values, while
`pp.sigma`, `pp.mu`, `pp.rho` create percent protected values. The second part of the
name is the parameter which is changed. For example, `eff.sigma` creates efficacy values for different
values of sigma. See details for a more complete description. Default for eff. functions is integration, default
for pp. functions is simulation.

### Usage

```
eff.sigma(mu, sigmas, COLORS = c("red", "green", "blue"), rho = 0, ...)
eff.mu(mu, factor = c(1/10, 1/3, 1/2, 1), COLORS = c("red", "green", "blue", "black"), sigma = 0.553, rho = 0, ...)
eff.rho(mu, sigma = 0.553, rho = c(0, 0.25, 0.5, 0.75, 1), COLORS = c("black", "blue", "green", "red", "black"), ...)
pp.sigma(mu, sigmas, COLORS = c("red", "green", "blue"), rho = 0, nsim = 10^5)
pp.mu(mu, factor = c(1/10,1/3,1/2,1), COLORS = c("red", "green", "blue", "black"), sigma =0.553, rho = 0, nsim = 10^5)
pp.rho(mu, sigma = 0.553, rho = c(0, 0.25, 0.5, 0.75, 1), COLORS = c("black", "blue", "green", "red", "black"), nsim = 10^5)
```

### Arguments

|  |  |
| --- | --- |
| `mu` | a vector of values of the mean of the log10 antibody |
| `factor` | a vector of values for defining the means of the second and third component (see details and warnings) |
| `COLORS` | colors for the plots, the ith color corresponds to the ith value of the parameter which is changing |
| `sigmas` | a vector of values of the standard deviation of the log10 antibody |
| `sigma` | a single value for sigma |
| `rho` | correlation vector (of length one for .sigma and .mu functions) of the log10 antibody, negative values not allowed |
| `nsim` | number of simulations for hbpp function |
| `...` | additional parameters may be added to the `hbrr` function |

### Details

For `eff.sigma` and `pp.sigma` we change sigma over the one, two, and three component model.
For `eff.mu` and `pp.mu` we change the mean over the two and three component model. For `eff.mu`
and `pp.mu`
the factor parameter is associated with each level of the second and third component.
See `vignette("hbimdetails")` for details.
For `eff.rho` and `pp.rho` we change the correlation over the two and three component model; for the jth column of the
out2 and out3 matrices, all correlations are given by jth level of factor. Because these calculations may take
hours, we save the original calculations used in the paper as output data,
`deff.sigma`,
`deff.mu`,
`deff.rho`,
`dpp.sigma`,
`dpp.mu`, and
`dpp.rho`. These output data set may be accessed by the command data(). For example, to access
`deff.sigma` type `data(deff.sigma)`.

### Value

A list with items

|  |  |
| --- | --- |
| `out1` | response matrix for one component model, ith row corresponds to mu[i] and jth column corresponds to the jth level of the parameter which is changing |
| `col1` | colors corresponding to columns of out1 |
| `out2` | response matrix for two component model, ith row corresponds to mu[i] and jth column corresponds to the jth level of the parameter which is changing |
| `col2` | colors corresponding to columns of out2 |
| `out3` | response matrix for three component model, ith row corresponds to mu[i] and jth column corresponds to the jth level of the parameter which is changing |
| `col3` | colors corresponding to columns of out3 |
| `cparms` | input vector of parameter that changes, e.g., factor vector |
| `sigma` | input sigma |
| `rho` | input rho |

### Warning

Note to save computation time these functions do not check that all variance-covariance matrices used
in the internal fucntions are positive definite. If you get an error message you do not understand check to see if the variance-covariance matrix is
positive definite by checking the eigen values. For example, with sigma=1, rho=-.6, the 3 components model do not have a positive definite variance-covariance
matrix because there is a negative eigenvalue
(to see this run `eigen(make.v(3,-.6,1))` ).

### Author(s)

M.P. Fay

### See Also

`vignette("hbimdetails")`

---

[Package *hbim* version 0.9.5 Index]
