# Supplementary material for: Human Immunity and the Design of Multi-Component, Single Target Vaccines
Source: PLoS One. 2007 Sep 5;2(9):e850. doi: 10.1371/journal.pone.0000850 (PMC1952173; doi:10.1371/journal.pone.0000850)
Supplement: Software S1 — Multi-component, single target vaccine R program software package. The R package containing the model. Instructions for unzipping and installing this program are contained in the supplementary file Hbimdetails.pdf (0.60 MB ZIP) [file pone.0000850.s004.zip › hbim/html/equiv.ab.html]

R: Equivalent antibody calculations by Linear Interpolation

|  |  |
| --- | --- |
| equiv.ab {hbim} | R Documentation |

## Equivalent antibody calculations by Linear Interpolation

### Description

This function inputs two antibody by response curves and outputs values needed for
plots of equivalent antibody response.
This is called by other functions (`plotresp.equiv`, `plotresp.mix`).
It is not to be called directly. For that purpose use `equiv.increase`.

### Usage

```
equiv.ab(effab1, ab1, effab2, ab2, npts = 100)
```

### Arguments

|  |  |
| --- | --- |
| `effab1` | vector of responses for antibody 1 |
| `ab1` | vector of doses of antibody 1 |
| `effab2` | vector of responses for antibody 2 |
| `ab2` | vector of doses of antibody 2 |
| `npts` | number of points used in some output |

### Details

The function uses the `approx` function to do linear interpolation and find the needed
values.

### Value

A list containing:

|  |  |
| --- | --- |
| `abpts` | a vector of values of antibody dose |
| `abpts10` | antilog of abpts, i.e., abpts raised to tenth power |
| `equiv.eff2` | equivalent response of antibody 2 |
| `equiv.eff1` | equivalent response of antibody 1 |
| `equiv.ab1` | vector of antibody doses that correspond with equiv.eff1 |
| `x` | equiv.ab1-abpts |
| `y` | equiv.eff1 |

### See Also

`equiv.increase`

---

[Package *hbim* version 0.9.5 Index]
