# Supplementary material for: Human Immunity and the Design of Multi-Component, Single Target Vaccines
Source: PLoS One. 2007 Sep 5;2(9):e850. doi: 10.1371/journal.pone.0000850 (PMC1952173; doi:10.1371/journal.pone.0000850)
Supplement: Software S1 — Multi-component, single target vaccine R program software package. The R package containing the model. Instructions for unzipping and installing this program are contained in the supplementary file Hbimdetails.pdf (0.60 MB ZIP) [file pone.0000850.s004.zip › hbim/html/equiv.increase.html]

R: Calculate equivalent increase from two dose-response curves

|  |  |
| --- | --- |
| equiv.increase {hbim} | R Documentation |

## Calculate equivalent increase from two dose-response curves

### Description

This function takes two curves defined by vectors of x and y values and
calculates the equivalent increase in the x value at the response value for the
first curve at e1.

### Usage

```
equiv.increase(x1, y1, x2, y2, e1, xlog = TRUE)
```

### Arguments

|  |  |
| --- | --- |
| `x1` | x vector for first curve |
| `y1` | y vector for first curve |
| `x2` | x vector for second curve |
| `y2` | y vector for second curve |
| `e1` | vector of y responses of first curve for associating with output |
| `xlog` | TRUE if x values are log transformed, changes the output |

### Details

The function repeatedly uses the `approx` function to do linear interpolation.

### Value

A list with 5 components

|  |  |
| --- | --- |
| `a1` | vector of x values associated with e1 from first curve |
| `e2` | vector of y values associated with a1 from the second curve |
| `a2` | vector of x values associated with e2 from the second curve |
| `e1` | input vector for e1 |
| `equiv.increase` | vector of equivalent increases associated with e1 |

### Examples

```
data(deff.sigma)
D<-deff.sigma
equiv.increase(D$mu,D$out1[,2],D$mu,D$out2[,2],.5)
```

---

[Package *hbim* version 0.9.5 Index]
