# Supplementary material for: Human Immunity and the Design of Multi-Component, Single Target Vaccines
Source: PLoS One. 2007 Sep 5;2(9):e850. doi: 10.1371/journal.pone.0000850 (PMC1952173; doi:10.1371/journal.pone.0000850)
Supplement: Software S1 — Multi-component, single target vaccine R program software package. The R package containing the model. Instructions for unzipping and installing this program are contained in the supplementary file Hbimdetails.pdf (0.60 MB ZIP) [file pone.0000850.s004.zip › hbim/html/hbim-internal.html]

R: Internal functions not be to called directly

|  |  |
| --- | --- |
| hbim-internal {hbim} | R Documentation |

## Internal functions not be to called directly

### Description

These are functions that are called by the `hbrr` and `hbpp`
functions. They integrate 1, 2 or 3 component models or integrate by simulation. Special functions
ending in .rhoeq1 are for when rho equals 1.

### Usage

```
hbpp.integrate1(MU, V, A = 1, RP = 0.1, ...)
hbpp.integrate2(MU, V, A = c(1, 1), RP = 0.1, ...)
hbpp.integrate3(MU, V, A = c(1, 1, 1), ...)
hbpp.simulate(MU, V, A, RP = 0.1, NSIM = 10^5)
hbrr.integrate1(MU, V, A = 1, ...)
hbrr.integrate2(MU, V, A = c(1, 1), ...)
hbrr.integrate2.rhoeq1(MU, V, A = c(1, 1), ...)
hbrr.integrate3(MU, V, A = c(1, 1, 1), ...)
hbrr.integrate3.rhoeq1(MU, V, A = c(1, 1, 1), ...)
hbrr.simulate(MU, V, A, NSIM = 10^4)
```

### Arguments

|  |  |
| --- | --- |
| `MU` | mean vector of the log10 dose |
| `V` | variance matrix of the log10 dose |
| `A` | vector of slope parameters in the Hill model, one for each component |
| `NSIM` | number of simulations |
| `RP` | protection bound, an individual is protected if relative risk is greater than RP |
| `...` | additional parameters to pass to the `integrate` function |

### Value

a numeric value of the expected relative risk or percent protected.

### Author(s)

M.P. Fay

### References

Saul, Fay (2007).

---

[Package *hbim* version 0.9.5 Index]
