# Supplementary material for: Human Immunity and the Design of Multi-Component, Single Target Vaccines
Source: PLoS One. 2007 Sep 5;2(9):e850. doi: 10.1371/journal.pone.0000850 (PMC1952173; doi:10.1371/journal.pone.0000850)
Supplement: Software S1 — Multi-component, single target vaccine R program software package. The R package containing the model. Instructions for unzipping and installing this program are contained in the supplementary file Hbimdetails.pdf (0.60 MB ZIP) [file pone.0000850.s004.zip › hbim/html/hbim-package.html]

R: Hill/Bliss Independence Model for Multicomponent Vaccines

|  |  |
| --- | --- |
| hbim-package {hbim} | R Documentation |

## Hill/Bliss Independence Model for Multicomponent Vaccines

### Description

Calculate expected relative risk and proportion protected assuming normally distributed log10 transformed antibody dose for several component vaccine. Uses Hill models for each component which are combined under Bliss independence.

### Details

|  |  |
| --- | --- |
| Package: | hbim |
| Type: | Package |
| Version: | 0.9.5 |
| Date: | 2007-06-05 |
| License: | GPL |

The hbim package allows users to reproduce plots and calculations for Saul and Fay (2007). See `vignette("hbimdetails")`.

### Author(s)

M.P. Fay,
Maintainer: Michael Fay <mfay@niaid.nih.gov>

### References

Saul, A. and Fay, M.P. (2007). Multi-component, single target vaccines:
design considerations derived from mathematical models.

---

[Package *hbim* version 0.9.5 Index]
