# Supplementary material for: Human Immunity and the Design of Multi-Component, Single Target Vaccines
Source: PLoS One. 2007 Sep 5;2(9):e850. doi: 10.1371/journal.pone.0000850 (PMC1952173; doi:10.1371/journal.pone.0000850)
Supplement: Software S1 — Multi-component, single target vaccine R program software package. The R package containing the model. Instructions for unzipping and installing this program are contained in the supplementary file Hbimdetails.pdf (0.60 MB ZIP) [file pone.0000850.s004.zip › hbim/html/hbrr.html]

R: Calculate expected relative risk or percent protected from Hill model with Bliss Independence

|  |  |
| --- | --- |
| hbrr {hbim} | R Documentation |

## Calculate expected relative risk or percent protected from Hill model with Bliss Independence

### Description

Assuming that the log10 transformed doses are normally distributed, we calculate the
expected relative risk (using `hbrr`) or percent protected (using `hbpp`)
from the Hill model using Bliss Independence. Numeric integration
is the default for up to three components for `hbrr`, while simulation is the default for
two or three components for `hbpp`.

### Usage

```
hbrr(mu, v, a = rep(1, length(mu)), simulate = FALSE, nsim = 10^4, ...)
hbpp(mu, v, a = rep(1, length(mu)), rp = 0.1, simulate = FALSE, nsim = 10^5, ...)
```

### Arguments

|  |  |
| --- | --- |
| `mu` | mean vector of the log10 dose |
| `v` | variance matrix of the log10 dose |
| `a` | vector of slope parameters in the Hill model, one for each component |
| `simulate` | estimation by simulation (TRUE) or numeric integration (FALSE) |
| `nsim` | number of simulations, ignored if simulate=FALSE |
| `rp` | protection bound, an individual is protected if relative risk is greater than rp |
| `...` | additional parameters to pass to the `integrate` function |

### Details

Although the package `adapt` can do multidimensional integration, we have written
specific functions to do this for up to 3 dimensions. This allows faster and more accurate integration.
The integration is done by repeated calls to the `integrate` function. The functions which do the
actual integration or simulation are internal functions which are not intended to be called by the user.
These internal functions are: for `hbrr`, when
simulate=FALSE, the function
calls one of either `hbrr.integrate1`, `hbrr.integrate2`, `hbrr.integrate2.rhoeq1`, `hbrr.integrate3`,
or `hbrr.integrate3.rhoeq1`
(for 1,2, or 3 component, with or without rho=1, taken from the size of the mu vector and dimension of the v matrix)
and when simulation=TRUE it calls `hbrr.simulate`. Similar functions exist for `hbpp`; however,
the `hbpp.integrate2` and `hbpp.integrate3` may have problems because of the discontinuity
in the integration function. That is why for two or three component models `hbpp.simulate` is used by default.

### Value

a numeric value of the expected relative risk or percent protected.

### Author(s)

M.P. Fay

### References

Saul, Fay (2007).

### Examples

```
## example of two dimensional integral
hbrr(c(.123,.432),matrix(c(1,.5,.5,1),2,2))
## faster but less accurate estimation by simulatin
hbrr(c(.123,.432),matrix(c(1,.5,.5,1),2,2),simulate=TRUE,nsim=10^4)
```

---

[Package *hbim* version 0.9.5 Index]
