# Supplementary material for: Human Immunity and the Design of Multi-Component, Single Target Vaccines
Source: PLoS One. 2007 Sep 5;2(9):e850. doi: 10.1371/journal.pone.0000850 (PMC1952173; doi:10.1371/journal.pone.0000850)
Supplement: Software S1 — Multi-component, single target vaccine R program software package. The R package containing the model. Instructions for unzipping and installing this program are contained in the supplementary file Hbimdetails.pdf (0.60 MB ZIP) [file pone.0000850.s004.zip › hbim/html/irdata.html]

R: Immune Response data

|  |  |
| --- | --- |
| irdata {hbim} | R Documentation |

## Immune Response data

### Description

Data from literature.

### Usage

```
data(irdata)
```

### Format

A data frame with 574 observations on the following 16 variables.

`RecordNum`
:   a numeric vector

`Old.Reference`
:   a numeric vector

`Reference`
:   a numeric vector

`Vaccine.and.trial.group`
:   a factor with levels `(Pentacel +Recombivax) then Prevnar` `11 valent Pneumonococcal-DT Finnland` `11 valent Pneumonococcal-DT Israel` `11 valent Pneumonococcal-DT+alum Finnland` `11 valent Pneumonococcal-DT+alum Israel` `11 valent Pneumonococcal-DT+alum Philippines` `ACTHIB` `AP-YF` `AVA IM` `AVA SQ` `BERNA-YF` `Boostrix` `Boostrix + Poliorix` `Boostrix Polio (dTpa-IPV)` `DPaT-HVB-IPV/HIB mix` `DPaT-HVB-IPV+HIB separate` `DPaT-IPV/HiB mix` `DPaT-IPV+HiB separately` `DPT +Hib/HBV` `DPT +Hib + HBV` `DPTa` `DPTa/Pa` `DPTa5` `DT` `DTP-HB + Hib` `DTPa-HBV` `DTPa/IPV/PRP-TT +PncD/T11` `DTPa2` `DTPa3` `DTPa5` `DTPaHBV-IPV+Hib` `DTPaHBV-IPV+Hib, PRP` `DTPaHBV-IPV+Hib, PRP-TT` `DTPw-C` `DTPw-E` `DTPw/IPV/PRP-TT +PncD/T11 group 1` `DTPw/IPV/PRP-TT +PncD/T11 group 2` `DTPwHBV-Hib` `DTPwHBV + Hib(Separate)` `Engerix B` `GBS Ia-TT 15 ug` `GBS Ia-TT 3.75 ug` `GBS Ia 55 ug` `GBS IaTT 60 ug` `GBS Ib-TT 15.75 ug` `GBS Ib-TT 3.94 ug` `GBS Ib-TT 63 ug` `GBS Ib 53 ug` `GBS II-TT` `GBS II-TT 14.3 ug` `GBS II-TT 3.6 ug` `GBS II-TT 57 ug` `GBS II-TT/III-TT` `GBS III-TT` `GBS III-TT 14.5 ug` `GBS III-TT 3.6 ug` `GBS III-TT 58 ug` `GBS III 50 ug` `GBS V-CRM197` `GBS V-TT` `GBS V-TT 2.4/1.1 ug` `GBS V-TT 38.5/17 ug` `GBS V-TT 9.6/4.3 ug` `H5N1Influenza 45ug` `H5N1Influenza 7.5ug` `H5N1Influenza 90ug` `H5N1Influenza 15ug` `H5N1Influenza Placebo` `Havrix+Engerix B` `HBV/Pentavax` `Heptavalent Pneumonococcal-CMR197` `Heptavalent Pneumonococcal-OMPC` `Heptvalent Pneumonococcal-CMR197` `Hiberix` `HibTITER` `HibTITER (PRP-CRM197)` `Infantrix-IVP+Hib` `Infantrix-IVP+Hib+Previnar` `Infantrix + Engerix separate` `Infantrix/Engerix Mixed` `Infantrix/Engrerix Mixed` `IPV-mkc` `IPV-vero` `Lyme OspA 15ug` `Lyme OspA 30ug` `LYMErix (OpsA)` `MCV4-DT` `Menactra Meningococcal PS-DT (MCV-4)` `Mencevax ACWY` `Meningitec (Menc-CRM197)` `Menomune Meningococcal PS`  `Menomune Meningococcal PS (PSV-4)` `Octavalent Pneumonococcal-DT` `Octavalent Pneumonococcal-TT` `Oka/Merck varicella 16K PFU +M-M-R` `Oka/Merck varicella 50K PFU +M-M-R` `OpsA Lyme Disease` `Orimmune` `OspA Lyme` `Pentacel + Prevnar +Recombivax` `Pentavax` `Revaxis (Td-IPV)` `RKI-YF` `RSV PFP3` `Tripedia` `Tripedia-Orimune-HibTITER` `Twinrix` `Twinrix adult` `Twinrix pediatric` `Typhoid/HAV` `Varilrix + M-M-R`

`Carrier.for.conjugate.vaccines`
:   a factor with levels  `CRM197` `Diphtheria toxoid` `OMPC` `Tetanus protein` `Tetanus toxoid`

`Age.in.yrs.at.first.vaccination`
:   a factor with levels `0.12` `0.17` `0.17-0.5` `0.25` `0.5` `1` `1-12` `1 -2` `1.5` `11-18` `12-15` `15-18` `15-70` `16-65` `17-72` `18-32` `18-39` `18-40` `18-45` `18-50` `18-60` `18-64` `19-52` `19-56` `19-57` `19-64` `19-70` `19-83` `2` `2-5` `20-45` `20-60` `20-61` `21-60` `3` `4` `4-14` `40-70` `5-16` `65-83`

`Dose.schedule.in.weeks`
:   a factor with levels `0` `0, 2` `0, 2, 4` `0, 26` `0, 26/0, 4, 26` `0, 4` `0, 4, 10` `0, 4, 26` `0, 4, 52` `0, 4, 8` `0, 4, 8, 52` `0, 52` `0, 52, 104` `0, 6, 13` `0, 8` `0, 8, 16` `0, 8, 18` `0, 8, 18`  `0, 8, 18, 220` `0, 8, 18, 270` `0, 8, 18, 320` `0, 8, 18, 44` `0, 8, 18, 56` `0, 8, 18, 60` `0, 8, 18, 70` `0, 8, 18; 4,12,22` `0, 8, 18; 4,12,22`  `0, 8, 39` `0, 9, 37`

`Num.Immunizations`
:   a numeric vector

`Endpoint.in.weeks.after.first.vaccine`
:   a numeric vector

`Antigen`
:   a factor with levels `1` `14` `18C` `19F` `23F` `3` `4` `5` `6B` `7F` `9V` `A` `AVA` `C` `DT` `F protein` `FHA` `FIM` `HAV` `HBs` `Hemagglutinin` `Ia-CPS` `Ib-CPS` `II-CPS` `III-CPS` `Measles` `MenC` `Mumps` `OspA` `Polio-1` `Polio-2` `Polio-3` `PRN` `PRP` `PRP*` `PT` `Rubella` `TT` `V-CPS` `Varicella` `Vi` `W135` `Y` `YF`

`Units`
:   a factor with levels  `EL.U/mL` `EU` `HI` `IU` `mIU` `ng` `SBA` `ug`

`GMT`
:   a numeric vector

`GMT.95.pct.interval.low.limit`
:   a numeric vector

`GMT.95.pct.interval.high.limit`
:   a numeric vector

`n`
:   a numeric vector

`Fold.Range`
:   a numeric vector

### Source

See `data(refs)` for references

### Examples

```
data(irdata)
irdata[1,]
```

---

[Package *hbim* version 0.9.5 Index]
