# Supplementary material for: Human Immunity and the Design of Multi-Component, Single Target Vaccines
Source: PLoS One. 2007 Sep 5;2(9):e850. doi: 10.1371/journal.pone.0000850 (PMC1952173; doi:10.1371/journal.pone.0000850)
Supplement: Software S1 — Multi-component, single target vaccine R program software package. The R package containing the model. Instructions for unzipping and installing this program are contained in the supplementary file Hbimdetails.pdf (0.60 MB ZIP) [file pone.0000850.s004.zip › hbim/html/make.v.html]

R: Make Exchangeable Variance Matrix

|  |  |
| --- | --- |
| make.v {hbim} | R Documentation |

## Make Exchangeable Variance Matrix

### Description

Not to be called directly. Used by `eff.sigma`,
`eff.mu`,
`eff.rho`,
`pp.sigma`,
`pp.mu`, and
`pp.rho`.

### Usage

```
make.v(n, r, sig2)
```

### Arguments

|  |  |
| --- | --- |
| `n` | dimension of variance matrix |
| `r` | correlation |
| `sig2` | variance |

### Value

An variance-covariance matrix, with all diagonal elements equal and all off diagonal elements equal.

---

[Package *hbim* version 0.9.5 Index]
