# Supplementary material for: Human Immunity and the Design of Multi-Component, Single Target Vaccines
Source: PLoS One. 2007 Sep 5;2(9):e850. doi: 10.1371/journal.pone.0000850 (PMC1952173; doi:10.1371/journal.pone.0000850)
Supplement: Software S1 — Multi-component, single target vaccine R program software package. The R package containing the model. Instructions for unzipping and installing this program are contained in the supplementary file Hbimdetails.pdf (0.60 MB ZIP) [file pone.0000850.s004.zip › hbim/html/plotlogm.resp.html]

R: Plot Hill/Bliss Independence Model Data.

|  |  |
| --- | --- |
| plotlogm.resp {hbim} | R Documentation |

## Plot Hill/Bliss Independence Model Data.

### Description

These functions take data output calculated from the data generating functions (see details)
and plot either: the mean of the log transformed antibody doses by the response (`plotlogm.resp`),
equivalent increase in antibody plots (`plotresp.equiv`), or response
of one component versus a mixture (for details see `vignette("hbimdetails")`).

### Usage

```
plotlogm.resp(D, YLAB = "Efficacy", YLIM = c(0, 1), XLIM = c(-2, 2),TITLE="")
plotresp.equiv(D, XLIM = c(0, 1), YLIM = c(1, 100), RLAB = "Efficacy of", bounds= XLIM,TITLE="")
plotresp.mix(D, RLAB = "Efficacy of", XYLIM = c(0, 1),TITLE="")
```

### Arguments

|  |  |
| --- | --- |
| `D` | data, see details |
| `YLAB` | y label |
| `YLIM` | range of y axis |
| `XLIM` | range of x axis |
| `RLAB` | response label, currently use only either "Efficacy of" or "% Protected by" |
| `bounds` | bounds on response of second antibody curve, see `vignette("hbimdetails")` |
| `XYLIM` | range of both x and y axes |
| `TITLE` | title of plot |

### Details

The following functions create data sets for plotting:
`eff.sigma`,
`eff.mu`,
`eff.rho`,
`pp.sigma`,
`pp.mu`,
`pp.rho`.
These functions plot that data. For details see `vignette("hbimdetails")`.

### Value

Plots

---

[Package *hbim* version 0.9.5 Index]
