# Supplementary material for: Human Immunity and the Design of Multi-Component, Single Target Vaccines
Source: PLoS One. 2007 Sep 5;2(9):e850. doi: 10.1371/journal.pone.0000850 (PMC1952173; doi:10.1371/journal.pone.0000850)
Supplement: Software S1 — Multi-component, single target vaccine R program software package. The R package containing the model. Instructions for unzipping and installing this program are contained in the supplementary file Hbimdetails.pdf (0.60 MB ZIP) [file pone.0000850.s004.zip › hbim/html/refs.html]

R: Reference list

|  |  |
| --- | --- |
| refs {hbim} | R Documentation |

## Reference list

### Description

Each reference is one long character string. See `data(irdata)` for data from each reference.

### Usage

```
data(refs)
```

### Format

The format is:
Factor w/ 50 levels (the 50 references)

### Examples

```
data(refs)
refs[1]
```

---

[Package *hbim* version 0.9.5 Index]
